# The KLF4/Galectin-3 cascade is a key determinant of tubular cell death and acute kidney injury

**DOI:** 10.7150/ijbs.110790

**Published:** 2025-09-20

**Authors:** Lishan Liu, Fangxu Chen, Kang Liu, Feng Xu, Ruihua Shen, Juanjuan Jiang, Fang Lu, Jingfeng Zhu, Simeng Liu, Lin Wu, Ao Bian, Jamie R Privratsky, Steven D. Crowley, Lianmin Chen, Changying Xing, Yanggang Yuan, Zhimin Huang, Huijuan Mao, Jiafa Ren

**Affiliations:** 1 Department of Nephrology, the First Affiliated Hospital of Nanjing Medical University, Nanjing Medical University, Nanjing, China.; 2 Department of Anesthesiology, Duke University Medical Center, Durham, North Carolina, USA.; 3 Division of Nephrology, Department of Medicine, Duke University Medical Center, Durham, NC 27710, USA.; 4 Department of Cardiology, the First Affiliated Hospital of Nanjing Medical University, Nanjing Medical University, Nanjing, China.

**Keywords:** KLF4, Galectin-3, acute kidney injury, tubular cell death, inflammation.

## Abstract

Clinically, acute kidney injury (AKI) stems from a diverse array of causes including ischemia, exposure to nephrotoxic agents, or sepsis. Renal tubular cells are particularly vulnerable and often sustain the most significant damage during AKI. This raises the question of whether there exists a common pathophysiological mechanism or pathway in renal tubular cells that underlies the development of AKI. We observed that tubular Galectin-3 is significantly up-regulated in four AKI mouse models and its tissue expression shows a positive correlation with tubular injury in human kidneys affected by AKI. The urinary Galectin-3 levels were markedly elevated in a cohort of patients with AKI and these levels correlated with the severity of kidney dysfunction. Based on predictions from bioinformatic analysis and JASPAR database, ChIP-PCR and luciferase-reporter assays demonstrated the direct binding of the transcription factor KLF4 to a specific sequence in the Galectin-3 gene promoter. Furthermore, mice with proximal tubular-specific deletion of KLF4 exhibited reduced kidney injury and inflammation, along with lower Galectin-3 expression in both cisplatin and ischemia-reperfusion-induced AKI. Targeting the KLF4/Galectin-3 axis with Kenpaullone and GB1107 confirmed protective effects against cisplatin-induced cell death and acute kidney injury, respectively. Our study highlights the KLF4/Galectin-3 pathway as a key mediator in the pathogenesis of AKI. Disrupting this signaling pathway may provide a promising therapeutic approach for the treatment of AKI.

## Introduction

Acute kidney injury (AKI) is a serious clinical condition characterized by a rapid deterioration in renal function 1, 2. The pathophysiology of AKI primarily involves hypoxia, oxidative stress, and activation of the innate immune system, all of which lead to renal tubular cell death. Renal tubular cells constitute two thirds of the cells of the kidney. They are often the primary vulnerable targets and sustain the most significant damage during AKI 3-5. Moreover, damaged tubules frequently initiate and orchestrate the inflammatory response in the renal microenvironment by multiple mechanisms, including secreting various chemokines and cytokines 6, 7. Clinically, AKI is a heterogeneous syndrome that can arise from various causes, including ischemia, nephrotoxic agents, and sepsis 8. This raises the question of whether a common pathophysiological mechanism or signaling pathway in renal tubular cells contributes to the development of AKI. The current KDIGO definition of AKI relies on the measurement of serum creatinine (Scr) or urine output, neither of which are markers of tubular injury but rather reflect an abrupt decrease in glomerular filtration 9, 10. Although tubular 'damage' biomarkers for acute kidney injury, such as NGAL and KIM-1, are currently available, each has its own limitations 11. Our study aims to develop alternative noninvasive 'damage' biomarkers as surrogates to assess, predict, and monitor the development and progression of AKI in patients. To address this, we employed four distinct mouse models of AKI to conduct a single-cell sequencing analysis. Our findings revealed that Galectin-3 in proximal tubular cells (PTs) is commonly significantly up-regulated across all these models. Galectin-3, a β-galactoside-binding lectin, has been found as a pleiotropic modulator of fibrosis and inflammation 12. However, the mechanisms underlying the regulation of Galectin-3 and its role in modulating cell death and AKI remain incompletely understood. A combination of *in vitro* and *in vivo* analyses first revealed proximal tubular Galectin-3 was positively transactivated by the transcription factor KLF4. KLF4 known as a Yamanaka transcription factor, is responsible for cell reprogramming and pluripotency maintenance. KLF4 signaling has been implicated in the pathogenesis of various kidney diseases. For instance, endothelial KLF4 improves renal function and enhances the benefits of statins in ischemic acute kidney injury by decreasing inflammatory response 13. Macrophage KLF4 exhibits a renoprotective function during fibrogenesis by suppressing TNF-α-induced renal injury 14. Here, we demonstrate that urinary Galectin-3 has the potential to serve as a noninvasive biomarker, and targeting the KLF4/Galectin-3 axis with Kenpaullone and GB1107 showed protective effects against cisplatin-induced cell death and acute kidney injury, respectively, suggesting a promising therapeutic approach for AKI.

## Materials and Methods

### Human urine and kidney biopsy samples

Human kidney specimens were obtained from diagnostic renal biopsies conducted at the First Affiliated Hospital of Nanjing Medical University. Paracancerous normal kidney tissues obtained from patients with renal carcinoma who underwent nephrectomy were utilized as the normal controls. Paraffin-embedded sections of human kidney biopsies were prepared using a standard procedure and subsequently subjected to staining with the Galectin-3 (Abcam, cat. ab76245) and KLF4 antibody (R&D, cat. AF3640).

Human urine samples were obtained from the First Affiliated Hospital of Nanjing Medical University. Some urine samples were also collected from healthy volunteers. All the studies involving human samples were approved by the Ethic Committee on Human Subjects of the First Affiliated Hospital of Nanjing Medical University (2018-SR-080).

### Human galectin-3 ELISA and western blot analysis

The supernatant was collected after centrifuging urine at 3000×g for 15 minutes at 4°C. Human urinary Galectin-3 levels (MultiSciences Biotech, cat: EK1126, China) were measured according to the manufacturer's specified assay procedures.

The supernatant of urine was mixed with 5× loading buffer (Fudebio, cat: FD006, China), thoroughly mixed and boiled at 100 °C for 10 minutes before being subjected to Western blot analysis.

### Mice

Mice with a floxed *KLF4* allele were ordered from Cyagen (cat: S-CKO-03285, Guangzhou, China, C57BL/6J background). We generated mice with specific deletion of KLF4 from epithelial cells in the proximal tubular (*PCK-Cre*^+^ KLF4^fl/fl^, PKO) by using the C57BL/6 *Pepck-Cre* mouse lines as described previously 7. All animals were born normal with the expected Mendelian frequency. Male mice aged 8 to 12 weeks old were randomly assigned to further experiments. Mice were housed and bred in the animal facilities at the Laboratory Animal Center of Nanjing Medical University, according to the guidelines of the Institutional Animal Care and Use Committee from Nanjing Medical University (IACUC-2103053).

### Animal models of AKI

Cisplatin-indcued AKI, Male KO mice and their control, wild-type (WT), littermates were injected intraperitoneally (i.p.) with 20 mg/kg cisplatin (cat: P4394, Sigma-Aldrich) to induce acute kidney injury. After 72 hours, the injured kidneys and blood were harvested for analysis.

IR-induced AKI, Mice were anesthetized with isoflurane, using 2-3% for induction and 1-2% for maintenance. To induce ischemia-reperfusion kidney injury, the right kidneys were removed, and the left renal pedicles of mice were clamped for 24 minutes (at 37℃-38℃) and then were reperfused within 1 minute. After 72 hours post-surgery, the injured kidneys and blood were harvested for analysis.

Nephrotoxic Serum (NTS)-induced AKI, mice received an i.p. injection of sheep IgG (250 μg/mouse) on day 0. Tail injection of sheep NTS (catalog PTX-001S, Probetex) was performed on day 5. On day 9, mice were euthanized, and blood and kidney tissues were harvested for further analysis 14.

Aristolochic acid (AA)-induced AKI, male mice were injected daily i.p. with 4 mg/kg AA (cat: 313-67-7, sigma) for five consecutive injections to induce kidney injury. Mice were euthanized, kidney tissues were harvested 1 day later for further analysis 7.

Scr was measured with Creatinine Assay kit (cat: E-BC-K188-M, Elabscience, Wuhan, China) and Blood urea nitrogen (BUN) was measured with Urea Assay kit (cat: EIABUN; Thermo Fisher Scientific, Waltham, MA) according to the manufacturer's instructions.

Male mice received GB1107 (cat: HY-114409, MedChemExpress)^15^, ^16^ or Kenpaullone (cat: HY-12302, MedChemExpress)^17^ at a dose of 5mg/kg per day, starting one day prior to cisplatin injection and continuing throughout the experiment.

### ScRNA-seq methods

#### Kidney Digestion and Cell Isolation

Our kidney digestion and cell isolation protocol has been previously described 7. the AA- or NTS- challenged condition involved combining kidneys from three AA- or NTS- challenged mice to minimize potential biological and technical variability of the model. After library preparation, samples were sequenced on a Novaseq 6000 (Illumina) S2 flow cell by the Duke Center for Genomic and Computational Biology Sequencing Core. Cell Ranger software from 10X Genomics was used to transform raw base call files into FASTQ files (cellranger mkfastq) and align reads with reference mouse genome (cellranger count). The scRNA-seq datasets for cisplatin (GSE197266) 18 and IR-induced (GSE139506) 19 AKI were downloaded from the Gene Expression Omnibus (GEO) database (http://www.ncbi.nlm.nih.gov/geo).

#### Normalization, sample integration and clustering

The Seurat package (5.1.0) was used to perform analysis on scRNA-seq data. To exclude low-quality cells, we kept all genes expressed in more than three cells and cells with at least 200 detected genes. Cells with unique gene counts more than 7500 or less than 200 were discarded. We also filtered out cells for which the percent of mitochondrial gene expression represented more than 75% of the total gene expression. Meanwhile, the DoubletFinder package was used to filter out doublets. The data was normalized by NormalizeData and ScaleData functions. Each cell's gene was scaled by a factor of 10,000 and log-transformed. A total of 2000 genes with the highest variability were identified. Next, samples from different group were combined using the FindIntegrationAnchors function, and batch effects were mitigated using the IntegrateData function. Subsequently, the gene expression matrix was reduced to the first 30 principal components, and a UMAP plot was generated to visualize the clusters at a resolution of 0.4 using the scRNAtoolVis package.

#### Unsupervised clustering and cell type annotation

The FindCluster function in Seurat was employed to identify cell clusters for each dataset. The FindMarkers function was used to compare gene expression across clusters. Differentially expressed genes were screened with the criteria of adjust p-value < 0.01 and log2 |fold change| > 1. Known markers for renal cell types were utilized to assign corresponding identities to each cluster (e.g., Slc34a1 and Lrp2 for proximal tubular cells, Umod and Slc12a1 for Loop of Henle cells, Calb1 and Slc12a3 for distal tubule cells, Nphs2 and Podxl for podocyte). We focused on the PT cell population, further subdividing it into Healty_s1 (Slc5a12, Adra1a), Healty_s2 (Slc22a30, Slc7a13), Healty_s3 (Slc22a30, Slc7a13, Bcat1), Repairing PT(Top2a), Injured PT (Myc, Havcr1, Krt20), Severe Injured PT (Krt20, Hspa1a), and Failed repair PT (Vcam1, Dcdc2a, Sema5a, Ccl2) 18, 19.

#### Gene set enrichment analysis

The UpSetR package was used to identify common upregulated and downregulated genes across the four AKI models. To assess the functional significance of the common upregulated genes, the clusterProfiler package was employed to conduct biological process (BP) enrichment analysis using Gene Ontology (GO). An adjusted p-value < 0.01 and a q-value < 0.25 were considered statistically significant.

#### Protein-protein interaction (PPI) network analysis

The STRING database (version 12.0, http://www.string-db.org/) was used to construct PPI network for the common upregulated genes. A required confidence threshold (combined score) of > 0.4 was applied, and the data were downloaded in CSV format. The network was subsequently constructed using Cytoscape software (version 3.9.1).

#### Transcription factor predicted by SCENIC analysis

Python (version 3.7) and the pySCENIC package (version 0.12.1) were used to assess the enrichment of transcription factors and the activity of regulons based on gene-motif ranking within a 10 kb region around the transcription start site. Motif annotation files were downloaded from the website https://resources.aertslab.org/cistarget. First, gene-gene co-expression modules between transcription factors and their putative targets were inferred using the GRNBoost2 method. Next, cis-regulatory motif discovery was employed to remove indirect targets from these modules. Finally, an enrichment score for the target genes of these regulons was calculated to analyze their activity. The ComplexHeatmap package (version 2.16.0) was then used to visualize regulon activity across different cells.

### Cell culture and treatment

Human renal proximal tubule cell line HK-2 were cultured in Dulbecco's Modified Eagle Medium/Nutrient Mixture F-12 (DMEM/F12, Basal Media) supplemented with 10% fetal bovine serum (FBS, AusGeneX, Australia) and 1% penicillin-streptomycin (Gibco, USA) at 37 °C with 5% CO2. Cells were seeded on six-well culture plates in DMEM/F12 containing 10% FBS. At 70%-80% confluence, cells were starved in serum-free medium for 12 hours and then treated with 25 μg/ml cisplatin for another 12 hours. siRNAs and plasmids (Shanghai Integrated Biotech Solutions, China) were transfected into HK2 cells using Lipofectamine 3000 (cat: L3000015, Invitrogen) according to the manufacturer's instructions.

### Terminal Deoxynucleotidyl Transferase-Mediated Digoxigenin-Deoxyuridine Nick-End Labeling Staining

Apoptotic cells in kidney sections or HK2 cells treated by cisplatin were determined using Terminal deoxynucleotidyl transferase-mediated deoxyuridine triphosphate (dUTP) nick-end labeling (TUNEL) staining (cat: A112-01, Vazyme Biotech, Nanjing) or Hoechst 33342/PI Kit (cat: CA1120, Solarbio, Beijing, China). Briefly, kidney cryosections or HK2 cells were fixed with 4% paraformaldehyde for 15 minutes, followed by permeabilization with 0.2% Triton X-100 in 1× PBS for 10 min at room temperature, the samples were then incubated with a TUNEL reaction mixture for 1 hour at 37 °C in a dark chamber; and the nuclei were labeled with DAPI. Positive staining with nuclear DNA fragmentation was detected by fluorescence microscopy. Ten fields were randomly selected from each section, and the average number of positive cells was counted.

### Annexin V/PI staining and flow cytometric analysis

Flow cytometric analysis was performed using the FITC Annexin V Apoptosis Detection Kit I (cat:556547, BD Pharmingen) to evaluate the percentage of apoptotic cells. Cells were harvested, washed twice with cold PBS, and resuspended in 100 μL of binding buffer. Cells were stained with 5 μL of annexin V-FITC for 15 minutes and 5 μL of PI for 5 minutes at room temperature in the dark, and then measured by laser flow cytometer (BECKMAN, USA) and quantified using FlowJo 10 software.

### RNA extraction and quantitative real-time PCR

Total RNA was extracted from cultured cells and kidney cortex using the FastPure® Cell/Tissue Total RNA Isolation Kit V2 (cat: RC112-01, Vazyme Biotech, Nanjing) according to the manufacturer's instructions. Following spectrophotometric quantification, cDNA was synthesized from the RNA using HiScript® III All-in-one RT SuperMix (cat: R333-01, Vazyme Biotech, Nanjing). Quantitative RT-PCR was performed using ChamQ SYBR qPCR Master Mix (cat: Q321-02, Vazyme Biotech, Nanjing) on a QuantStudio 7 real-time PCR system (Thermo, USA). GAPDH were used as internal controls, and the levels of target genes were calculated based on the 2^-ΔΔCT^ method.

### Western blot analysis

Kidney tissues were lysed with RIPA solution (cat: FD008, Fudebio) supplemented with a protease and phosphatase inhibitors cocktail (cat: FD1002, Fudebio) and 1mM PMSF (cat: FD0100, Fudebio) on ice for 30 minutes. Protein concentration was determined using the BCA Protein Assay Kit (KeyGEN, BioTECH, China) according to the manufacturer's instruction. Equal amounts of protein for each sample were loaded into SDS-PAGE gel and transferred onto PVDF membranes (cat: GVWP04700, Millipore). The membranes were blocked with 5% non-fat milk (Beyotime Biotechnology, China) in TBST buffer for 120 minutes, followed by probing with various primary antibodies overnight at 4℃. Afterward, the membranes were incubated with HRP-conjugated secondary antibodies at room temperature for 60 minutes. Bands were detected using an enhanced chemiluminescence detection system. Quantification was performed by measuring the intensity of the signals using the National Institutes of Health ImageJ software package and normalizing to the density of the corresponding GAPDH or α-tubulin bands in the same sample. Detailed information about the primary antibodies is provided in Supplementary Table 1.

### Histological analysis and immunohistochemistry staining

Kidneys from mice in all groups were fixed in 4% paraformaldehyde and embedded in paraffin. Paraffin sections were stained by the periodic acid-Schiff reagent, and the histologic changes were assessed by light microscopy. Renal damage, including necrotic tubules, loss of brush borders, tubule dilation, cast formation, tubular epithelial swelling, and vacuolar degeneration, was semiquantitatively scored. A score of zero represents an injury area <5%, whereas one, two, three, four, and five connote damage involving 5%-20%, 21%-40%, 41%-60%, 61%-80%, and >81% of the whole kidney area, respectively. The investigator, who was masked to experimental conditions, assessed injury percentage of at least ten random fields under the microscope (original magnification, X200), and a score was calculated for each mouse as previously described 7.

For immunohistochemistry staining (IHC), kidney sections (3 μm thickness) were deparaffinized, hydrated, and antigen-retrieved. Then, the sections were blocked with QuickBlock™ Blocking Buffer for Immunol Staining (cat:P0260, Beyotime Biotech-nology, China) at room temperature for 60 minutes, followed by incubation with anti-KIM-1 (cat: ab78494, Abcam, 1:2000), anti-NGAL (cat: ab216462, Abcam, 1:3000), anti-F4/80 (cat: ab300421, Abcam, 1:200), anti-Ly6G (cat: GB11229-100, Servicebio), anti-Galectin-3 (cat: ab76245, Abcam, 1:1000), anti-KLF4 (cat: A13673, Abclonal) overnight at 4℃ and then with HRP-conjugated secondary antibody (Proteintech) at room temperature for 60 minutes and the DAB substrate. Micrographs of the stained sections were captured by light microscopy and quantified using ImageJ. The number of F4/80-positive macrophages and Ly6G-positive neutrophils were counted from ten randomly selected fields in kidney for each sample under microscope (400×), and an average value for each section was calculated, as previously reported 20.

### Chromatin immunoprecipitation (ChIP)

ChIP assays were performed using the ChIP-IT High Sensitivity® kit (cat: 53040, Active Motif) according to the manufacturer's instructions. For *in vivo* ChIP assays, fresh renal tissue samples (250 mg) were chopped into small pieces with a razor blade and collected in ice-cold PBS. Formaldehyde was then added to the fixation solution to achieve a final concentration of 37%, and the samples were incubated at room temperature for 15 minutes with gentle rotation. The crosslinked chromatin was fragmented to 200-1000 bp by sonication at 30% amplitude, with a pulse of 30 seconds on and 30 seconds off for a total sonication “on” time of 30 minutes, all while keeping the samples on ice. Subsequently, equal amounts of samples, quantified by DNA concentration, were incubated with anti-KLF4 (cat: 11880-1-AP, Proteintech) or control IgG (cat: 2729, CST) along with magnetic protein G beads at 4℃ overnight with gentle rotation. After de-crosslinking and proteinase K treatment, the purified DNA was collected and subjected to standard PCR techniques.

### Dual-luciferase assay

The promoters of Lgals3 were cloned into the pGL4-basic vector. 293T cells at 70% confluence in 12-well plates were transfected with these plasmids, along with a Renilla luciferase vector (pRL-TK) as a control. After transfection, luciferase activity was measured using the Dual Luciferase Reporter Assay Kit (cat: DL101, Vazyme Biotech, Nanjing) according to the manufacturer's protocols. The relative Renilla luciferase activity was normalized to the firefly luciferase activity.

### Statistical analyses

All data were presented as mean ± s.e.m. Comparisons between groups were performed using 1-way analysis of variance followed by *post hoc* Tukey multiple comparisons test. The student's t test was used to compare 2 groups. All statistics were done in IBM SPSS v.20.0 and GraphPad Prism v.8.3.0. A difference was considered statistically significant if *p ˂ 0.05, **p ˂ 0.01, or ***p ˂ 0.001.

## Results

### Galectin-3 is recognized as a commonly upregulated candidate in injured tubular cells across multiple AKI models

**Figure 1:**
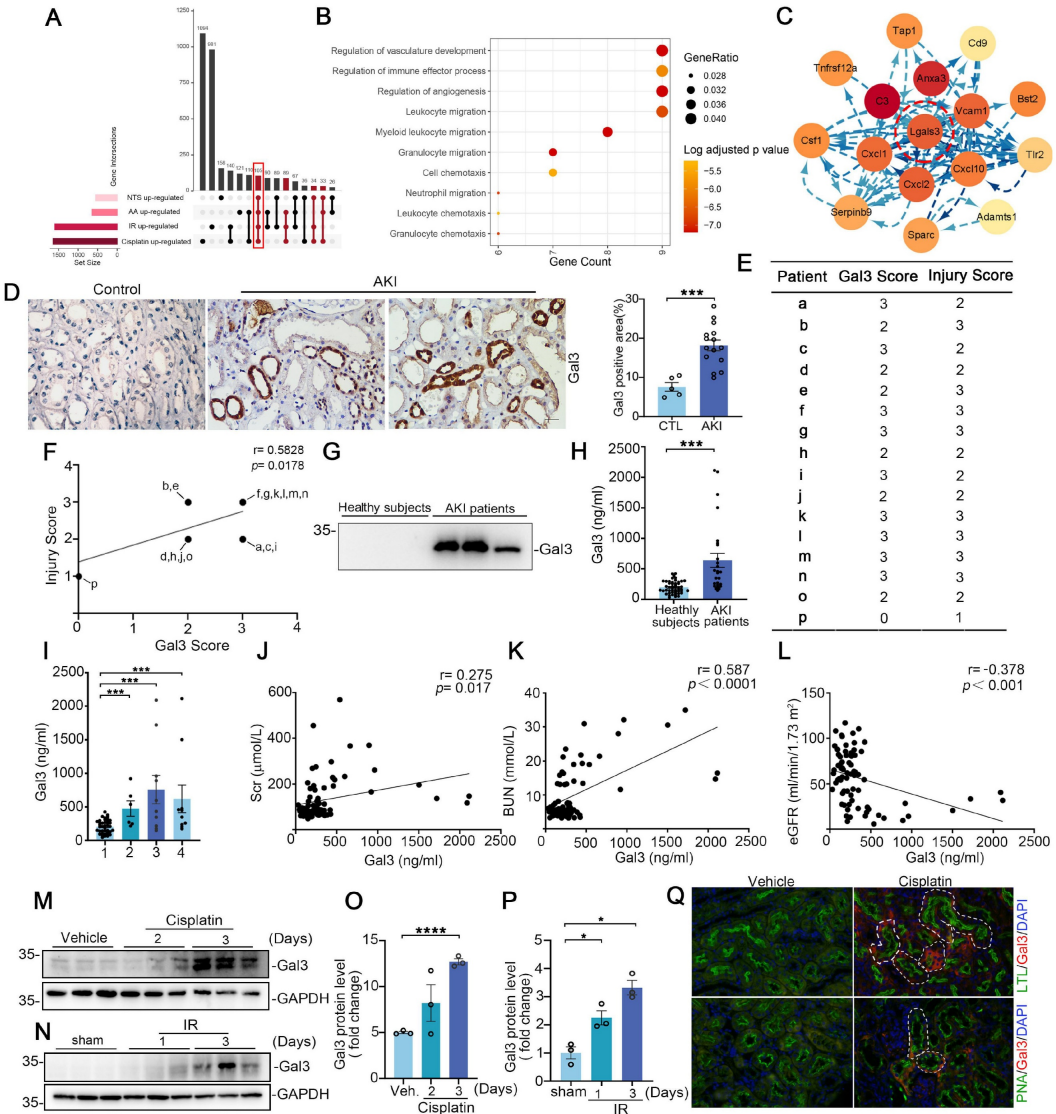
Tubular Lgals3 is identified as a key candidate gene across four models of acute kidney injury and associated with the severity of acute kidney injury in humans. (A) Upset plots showing up-regulated DEGs between PT cell clusters from these four AKI models. The bottom-left barplot showing the total high-interacting genes for each model. (B) GO functional analysis of shared genes from PT cell clusters across these four AKI models. (C) Protein-Protein Interaction network showing the top interacting genes among up-regulated DEGs. (D) Representative immunohistochemical staining images showing the expression of Galectin-3 in kidney tubular cells from patients with AKI. Scale bar = 50 μm. (E) The Galectin-3 score and tubular injury score for patients with acute kidney injury (n = 16). (F) The correlation between the two scores presented in (B) (n = 16). (G) Western blotting showing urinary Galectin-3 protein in healthy subjects and patients with AKI. (H) Graphic presentation shows urinary Galectin-3 protein levels in cohorts of patients with healthy subjects (n = 49) and AKI (n = 26). (I) Graphic presentation shows urinary Galectin-3 protein levels in different etiologies of AKI. 1, healthy subjects; 2, kidney disease; 3, septicopyemia-related AKI; 4, post-cardiac surgery-related AKI. (J-L) Linear regression analysis between urinary Galectin-3 levels and serum creatinine levels (J), BUN levels (K) or estimated glomerular filtration rate (eGFR) (L) in AKI patients and healthy subjects. (M-P) Western blot assays (M and N) and semiquantitative analysis (O and P) showing the abundance of Galectin-3 in the kidneys following cisplatin or ischemia reperfusion-induced AKI, as indicated by the groups (n = 3). (Q) Immunofluorescence co-staining of Galectin-3 with segment-specific tubular markers in cisplatin-challenged kidneys. The following segment-specific tubular markers were used: proximal tubule, lotus tetragonolobus lectin (LTL); distal tubule, peanut agglutinin (PNA). DEGs, differentially expressed genes; IR, ischemia-reperfusion; AA, aristolochic acid; NTS, nephrotoxic serum; Gal3, Galectin-3; AKI, acute kidney injury. IR, ischemia reperfusion. Data are presented as means ± SEM. *p˂0.05 or ***p˂0.001.

To investigate whether a common pathway or molecular protein is altered by various etiologies of AKI, we constructed single-cell sequencing datasets for two AKI models (aristolochic acid (AA)-induced and nephrotoxic serum (NTS)-induced) and combined them with two previously reported datasets (cisplatin-induced AKI, GSE197266, and ischemia-reperfusion-induced AKI, GSE161201) for further bioinformatics analysis. This approach aims to uncover the potential homogeneity of injury mechanisms across different etiologies of AKI. The cellular identity of each cluster was determined based on known cell type-specific markers (Supplementary Figure S1A). We then analyzed cells from the proximal tubular cell (PT) clusters, and identified a total of 105 up-regulated differentially expressed genes (DEGs) with FDR< 0.05, log2|FC| >1 between the disease group and the control group (Figure 1A); however, no down-regulated DEGs were identified across these four AKI models (Supplementary Figure S1B). The Gene Ontology (GO) cell compartment analysis indicated that these genes were markedly involved in the regulation of vascular development, immune response processes, cell killing and regulation of endocytosis (Figure 1B). To probe potential interactions among these 105 up-regulated DEGs, PPI networks were employed to construct functional gene associations. We used the STRING database and Cytoscape software and found *Lgals3*, *C3*, *Anxa3*, *Vcam1*, *Cxcl1*,* Cxcl2* and *Cxcl10* were key nodes (Figure 1C). Furthermore, we analyzed these nodal genes in PTs, which further sub-cluster four populations including failed repair PT, Injured PT, healthy_S1, healthy_S2 healthy_S3 or repairing PT subsets (Supplementary Figure S2A-D). We found that *Lgals3* was overrepresented in failed repair and/or injured PT cluster, especially in cisplatin and ischemia-reperfusion (IR)- induced AKI (Supplementary Figure S2A-B) compared to other genes.

### Galectin-3 levels in urine and kidney biopsies are associated with acute kidney injury

The expression and role of Galectin-3 has been most frequently reported in immune cells, while its functions and mechanisms in renal tubular cells remain poorly understood. Our immuno-histochemical staining of these acute kidney injury biopsies confirmed that induction of Galectin-3 was predominantly localized to kidney tubular cells and significantly increased compared with the controls, with little staining in the interstitium of diseased kidneys (Figure 1D). We blindly scored the levels of Galectin-3 and tubular injury in these human AKI kidney biopsies and found a positive correlation between them (Figure 1E-F). As Galectin-3 is reported to be a secreted protein, we hypothesized that increased renal Galectin-3 could lead to elevated levels in the urine. As shown in Figure 1G, Galectin-3 was readily detectable in the urine of patients with AKI, but not in healthy subjects. We further quantitatively measured urinary Galectin-3 levels in a cohort of 26 AKI patients and 49 healthy subjects using a specific ELISA. The demographic and clinical data of the patients are presented in Supplementary Table S2. As shown in Figure 1H, urinary Galectin-3 levels were elevated in patients with AKI, including those with septicopyemia, post-cardiac surgery and kidney diseases, compared to healthy subjects (Figure 1I). We also found urinary Galectin-3 was correlated with Scr, and BUN (Figure 1J-K). Inversely, Galectin-3 levels were negatively correlated with estimated glomerular filtration rate (eGFR) (Figure 1L). We further confirmed Galectin-3 protein expression in kidney tissue using in two rodent models of AKI. As shown in Figure 1M-P, Western blotting analysis revealed that expression of Galectin-3 was significantly increased in cisplatin-induced and ischemia reperfusion (IR)-induced injured kidneys. Immunofluorescence co-staining revealed that Galectin-3 was predominantly expressed in PTs in cisplatin-induced AKI models, with lower expression in distal tubular cells (Figure 1Q). Analysis of scRNA-seq datasets also confirmed that the average Galectin-3 expression in PTs across four AKI models is higher than in other cell clusters (Supplementary Figure S3A). These results indicated that proximal tubular Galectin-3 may play a critical role in the pathogenesis of AKI.

### Inhibition of proximal tubular Galectin-3 attenuates cell death and acute kidney injury

**Figure 2:**
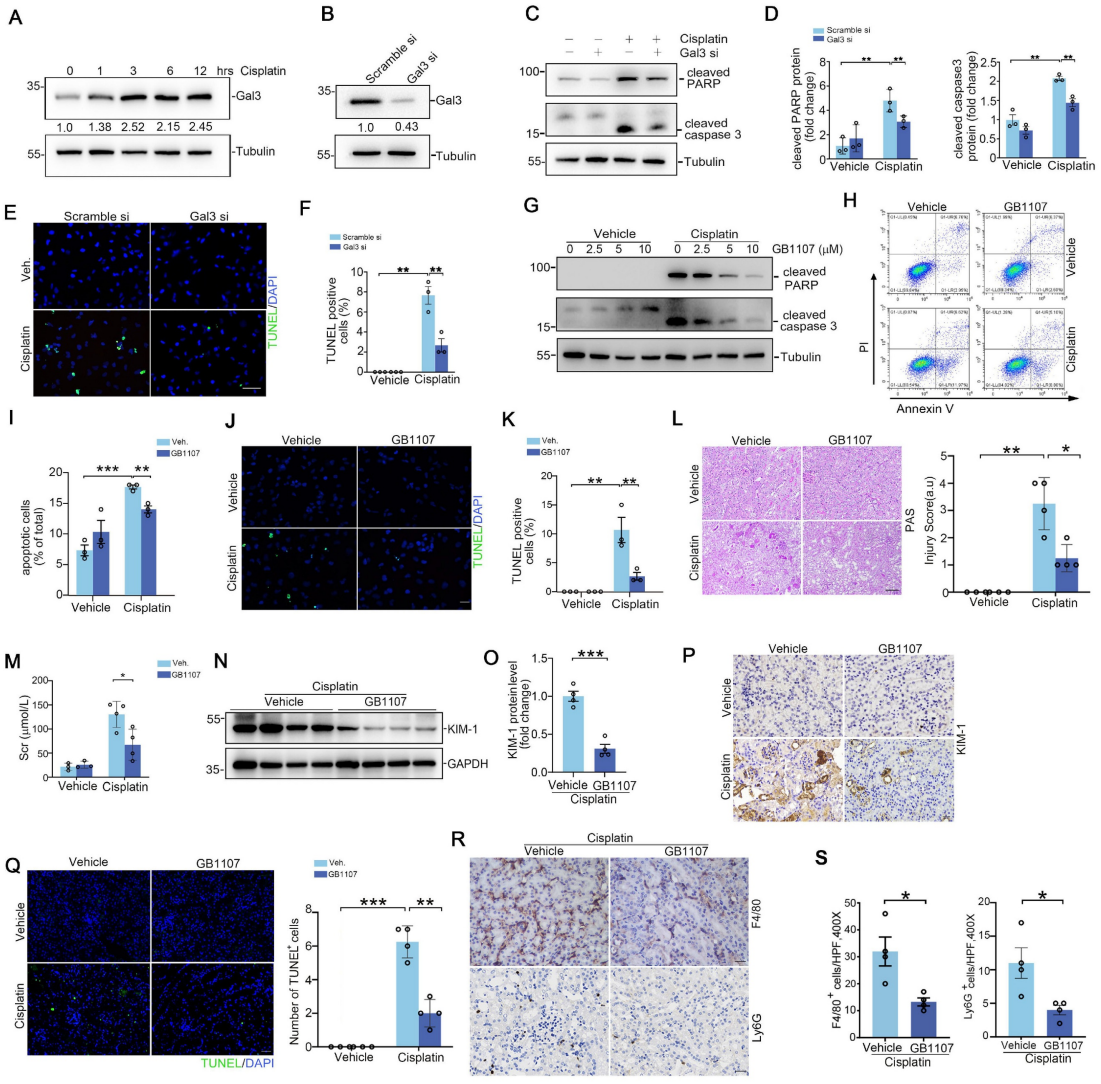
** Inhibiting Galectin-3 signaling attenuates kidney tubular cell death and acute kidney injury. (A)** Western blot showing Galectin-3 expression in HK2 cells treated with cisplatin (25 μg/ml) at different time points as indicated. **(B)** Galectin-3 expression at protein level in HK2 cells with Galectin-3 siRNA transfection. **(C)** Western blot for cleaved PARP protein and cleaved caspase 3 in HK2 cells transfected with scramble or Galectin-3 siRNA and followed by cisplatin (25 μg/ml) for 12 hours. **(D)** Semiquantitative analysis of cleaved PARP and cleaved caspase 3 protein levels from (C) (n = 3). **(E)** Representative images of TUNEL staining in HK2 cells treated with Galectin-3 or scramble siRNA, as indicated by the groups. Scale bar = 50 μm. **(F)** Quantification of TUNEL-positive cells per high-powered field in (E) (n = 3). **(G)** Western blot for cleaved PARP and cleaved caspase 3 protein in HK2 cells treated with GB1107 or vehicle, and followed by cisplatin (25 μg/ml) for 12 hours. **(H)** Representative flow cytometry plots analyzing GB1107 and vehicle-treated HK2 cells challenged w/o cisplatin, and stained with annexin-FITC and propidium iodide (PI) to identify cellular apoptosis. **(I)** Summary data quantifying apoptosis among different groups in (H) (n = 3). **(J)** Representative images of TUNEL staining in HK2 cells treated with GB1107 or vehicle, as indicated by the groups. Scale bar = 50 μm. **(K)** Quantification of TUNEL-positive cells per high-powered field in (J) (n = 3). **(L)** Kidney histology from the groups as shown by PAS staining and kidney pathology scores. Scale bar = 100 μm. **(M)** Serum creatinine levels in mice with different treatment (n = 4). **(N-O)** Western blots (N) and semiquantification (O) for KIM-1 protein from whole kidney tissue of vehicle and GB1107-treated mice with acute cisplatin nephropathy (n = 4). **(P)** Representative immunohistochemical staining for KIM-1 protein. Scale bar = 50 μm. **(Q)** Representative images and quantification analysis of TUNEL staining in kidney sections as indicated groups (n = 4). Scale bar = 50 μm. **(R)** Representative F4/80 and Ly6G stains of kidney sections from vehicle and GB1107-treated mice with cisplatin challenge. Scale bar = 50 μm. **(S)** Quantitative analysis for F4/80-positive macrophages and Ly6G-positive neutrophils presented in (R) (n = 4). Data are presented as means ± SEM. *p˂0.05, **p˂0.01, or ***p˂0.001.

To enhance the clinical relevance and applicability of our study, we employed the human proximal tubular epithelial cell line HK2 for our *in vitro* experiments. As shown in Figure 2A, Galectin-3 protein expression in HK2 was induced after cisplatin (25 μg/ml) exposure for 12 hours. The expression level of Galectin-3 protein in the Galectin-3 siRNA group showed a reduction of approximately 60% compared to that in the scramble siRNA group (Figure 2B). Western blot results indicated that, in scramble-transfected cells, cisplatin dramatically induced PARP and caspase 3 cleavage at 12 hours, an effect that was blunted in Galectin-3 siRNA group (Figure 2C-D). TUNEL-positive cells were largely decreased in cells transfected with Galectin-3 siRNA compared with those transfected with scramble siRNA, followed by cisplatin treatment (Figure 2E-F). Meanwhile, GB1107, a specific inhibitor of Galectin-3, was applied to inhibit Galectin-3 signaling pathway 21. As shown in Figure 2G, GB1107 attenuated the cisplatin-induced upregulation of protein levels of apoptosis marker cleaved PARP and cleaved caspase 3. Cells were co-stained with annexin V and propidium iodide (PI) and analyzed by fluorescence-activated sorting analysis to identify apoptotic cells. Flow cytometric analysis showed that the percentage of dead cells was markedly elevated after cisplatin treatment, whereas GB1107 treated cells exhibited less apoptosis after cisplatin treatment compared with scramble siRNA transfected-cells (Figure 2H-I). TUNEL staining also demonstrated the results shown above (Figure 2J-K).

To further confirm the findings *in vitro*, GB1107 was injected into CD-1 mice. Periodic acid-Schiff (PAS) stains of kidney tissue indicated that GB1107-treated kidneys showed relative preservation of kidney structure as confirmed by blinded injury scores (Figure 2L). Levels of Scr and renal Kim-1 protein expression were lower in the GB1107-treated group compared to the vehicle-treated group following cisplatin treatment (Figure 2M-P). In addition, the number of TUNEL^+^ cells in the GB1107-treated kidneys was significantly lower than that in the vehicle-treated group (Figure 2Q). To investigate whether inhibition of Galectin-3 with GB1107 could improve the inflammatory milieu of the kidney, we performed immunostaining on kidney tissues using antibodies against F4/80 and Ly6G to identify F4/80^+^ macrophages and Ly6G^+^ neutrophils, respectively. These immune cells exhibited no significant differences between vehicle- and GB1107-treated groups (Supplementary Figure S4A-B). However, they were markedly elevated in cisplatin-injured kidneys of vehicle-treated mice, whereas Galectin-3 inhibition with GB1107 significantly reduced their accumulation following cisplatin exposure (Figure 2R-S). This pattern of attenuated renal injury in the GB1107-treated cohort was recapitulated in mice subjected to IR-induced AKI (Supplementary Figure S5A-E). Thus, Galectin-3 signaling contributes to cisplatin and IRI-induced tubular cell death and inflammatory response, and its inhibition attenuates these effects.

### Galectin-3 is transcriptionally controlled by transcription factor KLF4, which promotes tubular cell death and inflammation

**Figure 3:**
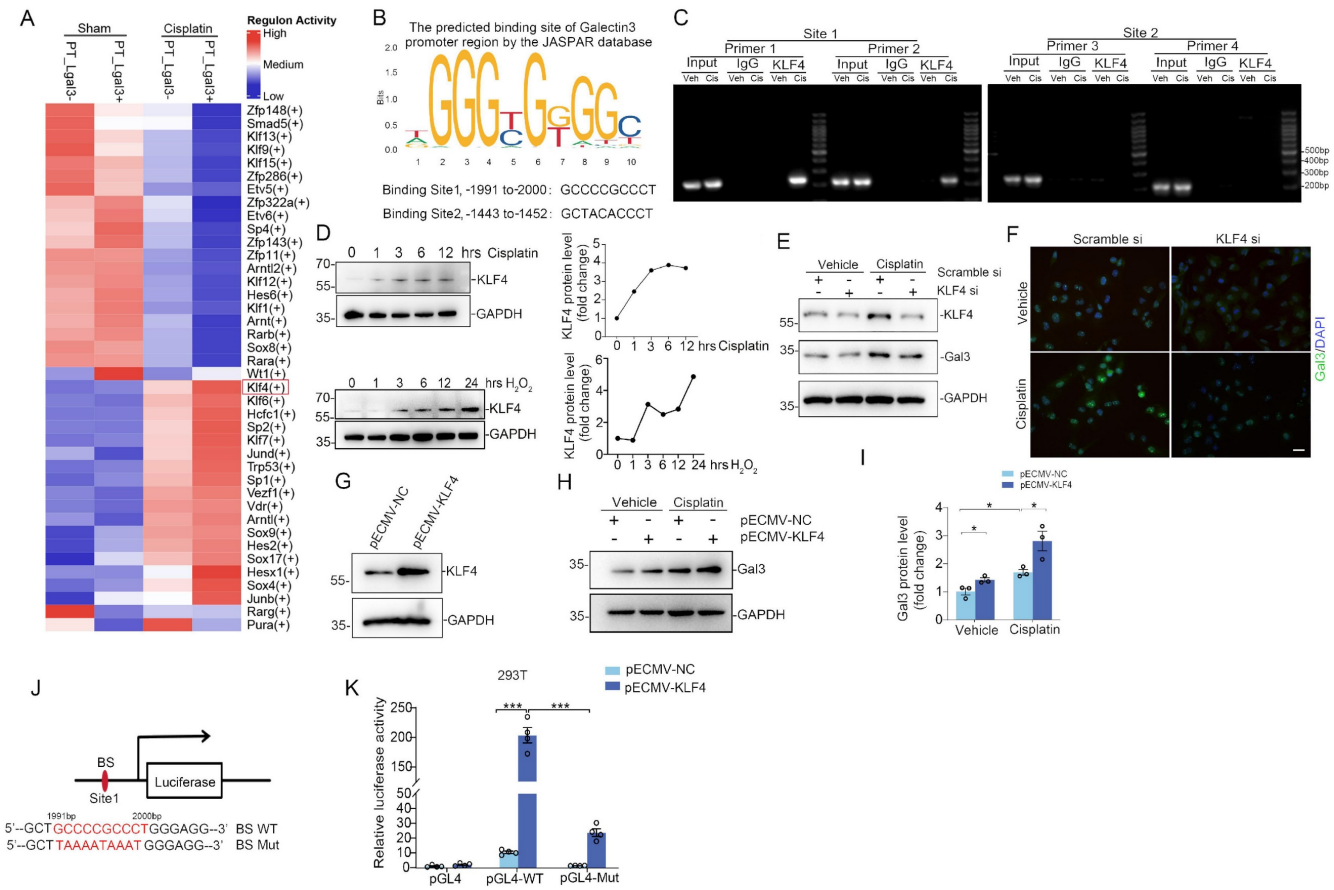
** The Galectin-3 promoter is bound and transactivated by KLF4. (A)** Heatmap analysis of transcription factor activity predicted by the SCENIC package for proximal tubular cells across the indicated groups. **(B)** The potential promoter sequences of Galectin-3 bound by the transcription factor KLF4, as predicted by the JASPAR database. **(C)** Chromatin immunoprecipitation-polymerase chain reaction (ChIP-PCR) assays showing PCR amplification of Galectin-3 chromatin corresponding to the region of the promoter (site1: nucleotides -1991 to -2000 and site2: -1443 to -1452 presented in (B)) immunoprecipitated with anti-KLF4 or with control IgG antibody from cisplatin-challenged kidneys. **(D)** Western blot and graphic presentation showing changes in KLF4 expression in HK2 cells treated with cisplatin (25 μg/ml) or H_2_O_2_ (500 mM) at different time points as indicated. **(E)** Western blot for KLF4 and Galectin-3 protein in HK2 cells transfected with scramble or KLF4 siRNA and followed by cisplatin (25 μg/ml) for 12 hours. One of the three independent experiments with identical results was shown. **(F)** Representative immunofluorescence staining images for Galectin-3 in HK2 cells transfected with scramble or KLF4 siRNA. Scale bar = 25 μm. **(G)** KLF4 protein expression in HK2 cells transfected with KLF4-OE plasmid. One of the three independent experiments with identical results was shown. **(H)** Galectin-3 protein levels in HK2 cells transfected with empty vector control or KLF4-OE plasmid followed by cisplatin treatment. **(I)** Semiquantitative analysis of Galectin-3 protein from (H) (n = 3). **(J)** Schematic illustration of Galectin-3 promoter reporter constructs containing the wild-type KLF4 binding sequences (BS WT) and the corresponding mutant sequences (BS Mut) used in luciferase assays. **(K)** Relative activation of WT and mutant Galectin-3 promoter by KLF4 in 293T cells. The luciferase activity of each group was normalized to that co-transfected with pECMV-NC and pgl4 plasmid (n = 4). Data are presented as means ± SEM. *p˂0.05 or ***p˂0.001.

The underlying mechanisms regulating Galectin-3 transcript in kidney tubular cells have not been clearly elucidated. To further explore the regulatory mechanisms of Galectin-3, transcription factor activities in Lgals3^+^ PT and Lgals3^-^ PT cells were analyzed. We observed a marked elevation of KLF4 activity in Lgals3^+^ PTs following cisplatin treatment compared to Lgal3^-^ PTs, while no significant alterations were observed between Lagal3^-^ PT and Lgals3^+^ PT from sham kidneys (Figure 3A). Moreover, the JASPAR database was utilized to predict potential transcription factor binding sites in the promoter region of Galectin-3 (http://jaspar.genereg.net). Consistently, KLF4 demonstrated the highest binding score among all the predicted transcription factors. Two potential KLF4 binding sites were identified upstream of the transcription start site (TSS): site 1 (-1991 bp to -2000 bp) and site 2 (-1443 bp to -1452 bp), for which two primer pairs were designed for each site based on the predictions from the JASPAR database (Figure 3B). ChIP-PCR assays were then performed to determine whether KLF4 directly binds to the promoter region of Galectin-3. According to ChIP-PCR analysis of kidney nuclei from sham and cisplatin-induced kidneys, it was found that KLF4 could directly bind to the Galectin-3 promoter region (site1, -1991 bp to -2000 bp) but not the region (site2, -1443 bp to -1452 bp) (Figure 3C), suggesting transcription factor KLF4 might be involved in the regulation of Galectin-3 through site 1. As shown in Figure 3D, KLF4 protein was induced under cisplatin or hydrogen peroxide (H_2_O_2_) treatment in a time-dependent manner in HK2. Knocking down KLF4 reduced the levels of Galectin-3 protein in HK2 cells assessed by Western blotting and immunofluorescence analysis (Figure 3E-F). Conversely, Overexpression of KLF4 by KLF4-overexpressing plasmid (KLF4-OE) led to an increase in the expression of Galectin-3 (Figure 3G-I) w/o cisplatin exposure. Consistently, a reporter plasmid containing a WT fragment spanning -1991 bp to -2000 bp of the Galectin-3 promoter was transactivated by KLF4. Furthermore, mutations within the -1991 bp to -2000 bp binding site abolished KLF4-dependent transactivation in 293T cells (Figure 3J-K). These results suggest KLF4 potentially regulates Galectin-3 expression though transactivating its promoter.

### Transcription factor KLF4 promotes tubular cell apoptosis by inducing Galectin-3 expression

**Figure 4:**
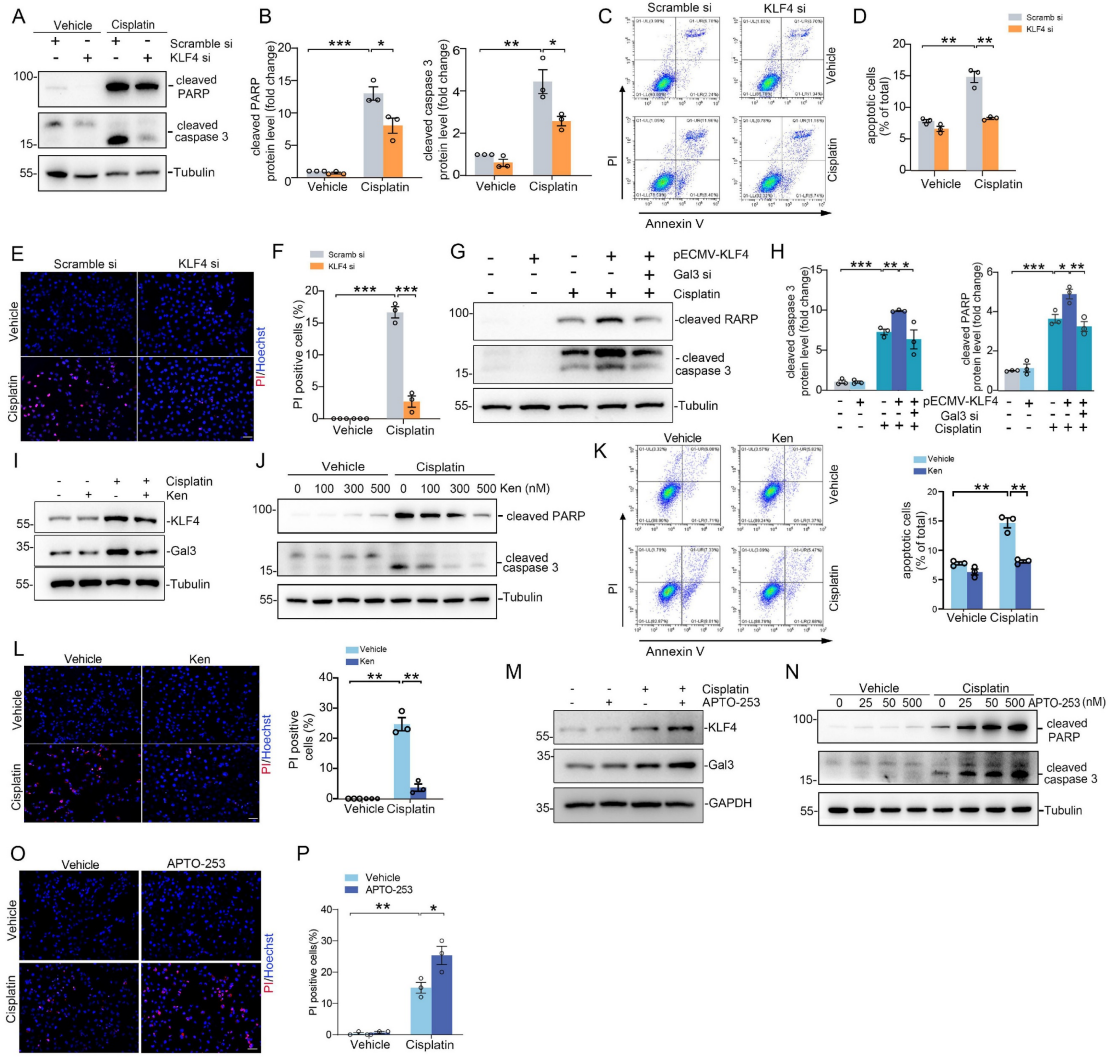
** Blocking the KLF4/Galectin-3 signaling cascade attenuates kidney tubular cell death. (A)** Western blot for cleaved PARP and cleaved caspase 3 protein in HK2 cells transfected with scramble or KLF4 siRNA followed by cisplatin exposure. **(B)** Semiquantitative analysis for cleaved PARP and cleaved caspase 3 protein from (A) (n = 3). **(C)** Representative flow cytometry plots analyzing KLF4 and scramble siRNA- treated HK2 cells, which were then treated with either vehicle or cisplatin. **(D)** Summary data quantifying apoptosis among different groups in (C) (n = 3). **(E-F)** Propidium Iodide (PI) staining assay (E) and quantitative analysis (F) of PI in HK2 cells among groups indicated. Scale bar = 50 μm. **(G)** Western blot for cleaved PARP and cleaved caspase 3 in HK2 cells with pECMV-KLF4 and/ or Galectin-3 siRNA followed by cisplatin treatment. **(H)** Semiquantitative analysis for cleaved PARP and cleaved caspase 3 protein levels from (G) (n = 3). **(I)** KLF4 and Galectin-3 protein expression in HK2 cells pretreated with Kenpaullone and followed by cisplatin (25 μg/ml) for 12 hrs. One of the three independent experiments with identical results was shown. **(J)** Western blot showing cleaved PARP and cleaved caspase 3 protein levels in HK2 cells pretreated with different concentrations of Kenpaullone followed by cisplatin exposure. **(K)** Representative flow cytometry plots and quantitative analyses of Kenpaullone- and vehicle-treated HK2 cells, as indicated by the groups (n = 3). **(L)** PI staining assay and quantitative analysis of PI in HK2 cells among groups indicated (n=3). Scale bar = 50 μm. **(M)** Western blots showing KLF4 and Galectin-3 protein expression in HK2 cells for the indicated group. One of the three independent experiments with identical results was shown. **(N)** Western blot analysis showing the levels of cleaved PARP and cleaved caspase 3 proteins in HK2 cells pretreated with various concentrations of APTO-253 before exposure to cisplatin. **(O-P)** PI staining assay (O) and quantitative analysis (P) of PI in HK2 cells among indicated groups (n=3). Scale bar = 50 μm. Ken, Kenpaullone; PI, Propidium Iodide. Data are presented as means ± SEM. *p˂0.05, **p˂0.01, or *** *p*˂0.001.

To investigate the effects of KLF4/Galectin-3 cascade on tubular cell survival, we first transfected HK2 cells with KLF4 siRNA and treated them with cisplatin. Western blotting analysis revealed that KLF4 knockdown diminished cisplatin-induced cleaved PARP and cleaved caspase 3 abundance compared with those in the scramble siRNA group (Figure 4A-B). Consistently, flow cytometric analysis showed that the percentage of dead cells, including early cell apoptosis (Annexin V^+^/PI^-^) and late cell apoptosis (Annexin V^+^/PI^+^), was markedly elevated after cisplatin treatment, whereas KLF4 siRNA-treated cells exhibited more apoptosis after cisplatin treatment compared with scramble siRNA groups (Figure 4C-D). PI staining analysis further confirmed the results obtained from Western blotting and flow cytometry analysis (Figure 4E-F). Inversely, overexpression of KLF4 by KLF4-OE plasmid enhanced cisplatin-induced cleaved PARP and cleaved caspase 3 production, whereas Galectin-3 knockdown significantly attenuated the effect of KLF4 on apoptosis in HK2 cells (Figure 4G-H).

Additionally, Kenpaullone, reported as a specific inhibitor of KLF4 22, 23, was applied to block KLF4 activation and subsequent Galectin-3 expression in HK2 cells (Figure 4I), without inhibiting KLF5, KLF6 or KLF15 protein expression (Supplementary Figure S6A). Western blot results demonstrated that cleaved PARP and cleaved caspase 3 were reduced in Kenpaullone-treated cells (Figure 4J). Flow cytometry analysis and PI staining assays further confirmed the results of Western blot analysis (Figure 4K-L). Conversely, Apto-253, an activator of KLF4 24, enhanced Galectin-3 expression (Figure 4M) and apoptosis in HK2 cells after cisplatin treatment, as assessed by Western blot analysis of cleaved PARP and cleaved caspase 3 (Figure 4N), as well as PI staining assays (Figure 4O-P). Apto-253 treatment did not increase the levels of other KLF family members, such as KLF5, KLF6 or KLF15 (Supplementary Figure S6B). Therefore, the KLF4/Galectin-3 signaling pathway is crucial for promoting tubular cell apoptosis.

### Specific ablation of KLF4 on proximal tubular cells protects against cisplatin- and IRI-induced acute kidney injury (AKI) in mice

**Figure 5:**
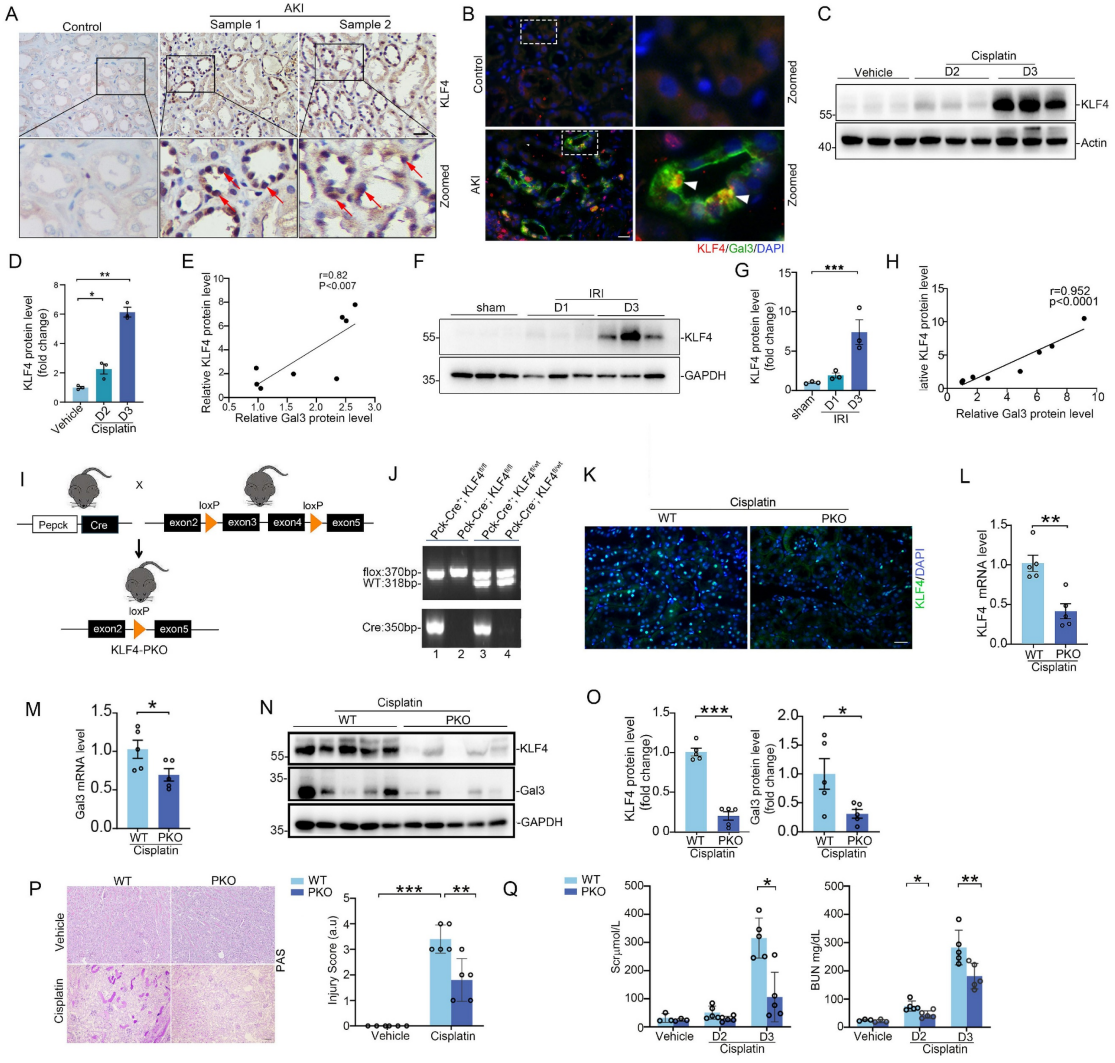
** KLF4 protein is induced in tubular cells from patients and mouse models with acute kidney injury, and deletion of KLF4 in proximal tubular cells attenuates cisplatin-induced AKI. (A)** Representative immunohistochemical staining images showing the expression of KLF4 in kidney tubular cells from patients with acute kidney injury (AKI). Red arrows indicating the KLF4 positive tubular cells. Scale bar = 50 μm. **(B)** Representative staining images showing colocalization of KLF4 and Galectin-3 proteins in kidney sections from patients with AKI. White arrow heads indicating double positive tubular cells. Scale bar = 50 μm. **(C-D)** Western blot assay (C) and semiquantitative analysis (D) showing the abundance of KLF4 protein in the mouse kidneys after cisplatin exposure at day 2 and 3 (n = 3). **(E)** Linear regression analysis of KLF4 and Galectin-3 expression levels in the kidneys of cisplatin mouse model. **(F-G)** Western blot assay (F) and semiquantitative analysis (G) showing the expression of KLF4 in the kidneys after IRI at day 1 and 3 (n = 3). **(H)** Linear regression analysis of KLF4 and Galectin-3 expression levels in the kidneys of IRI mouse model. **(I)** Strategy for generating mice with kidney proximal tubular-specific deletion of KLF4. **(J)** Genotyping the mice by PCR analysis of genomic DNA. **(K)** Representative immunofluorescence staining for KLF4 protein in WT and PKO kidney sections after cisplatin treatment. Scale bar = 50 μm. **(L-O)** KLF4 and Galectin-3 mRNA (L-M) and protein (N-O) expression levels in kidneys from WT and PKO mice following cisplatin exposure (n = 5). **(P)** Kidney histology from the groups as shown by PAS staining and kidney pathology scores (n = 5). Scale bar =100 μm. **(Q)** Serum creatinine and BUN among groups as indicated (n = 5). AKI, acute kidney injury; IRI, ischemia-reperfusion injury. Data are presented as means ± SEM. *p˂0.05, **p˂0.01, or *** *p*˂0.001.

To further characterize the tubular KLF4 expression in humans, immunohistochemistry assay revealed that KLF4 protein was markedly induced in damaged tubular cells from patients with AKI (Figure 5A). Immunofluorescence co-staining of KLF4 and Galectin-3 proteins confirmed a marked induction of both proteins in damaged tubular cells from kidney biopsies of patients with AKI (Figure 5B). Moreover, there was a positive correlation between Galectin-3 and KLF4 protein levels in both cisplatin- and IR-induced AKI rodent models (Figure 5C-H). Interestingly, we also observed a negative correlation between the levels of *KLF4 and LGALS3* mRNA expression with eGFR in tubulointerstitial region of patients' renal biopsy specimens across multiple datasets on Nephroseq (https://www.nephroseq.org; Supplementary Figure S7A-B). Because Galectin-3 is primarily localized in PT cells according to the analysis of single-cell sequencing datasets and immunochemical staining, we ablated *KLF4* on PT epithelial cell using a Cre-Lox gene targeting approach. *Pepck-Cre* has been verified in previous studies with a double-fluorescence reporter mouse 7. We therefore bred the Pepck-Cre mouse line with an *KLF4* flox line harboring loxp sites on either side of the coding region for the *KLF4* gene (Figure 5I). For our experiments, we used *Pepck-Cre*^+^ *KLF4*^flox/flox^ mice (PKO, Figure 5J, lane 1) and *Pepck-Cre^-^ KLF4*^flox/flox^ (WT, Figure 5J, lane 2). *KLF4* KO mice were phenotypically normal, with body weights similar to those of wild-type (WT) control littermates before cisplatin challenge or surgery (Supplementary Figure S8A). Both WT and PKO kidneys showed comparable architecture as assessed by PAS staining. Additionally, at baseline, PKO kidneys exhibited similar mRNA levels of AQP1, AQP2 and CDH16 compared to WT kidneys (Supplementary Figure S8B-C).

**Figure 6:**
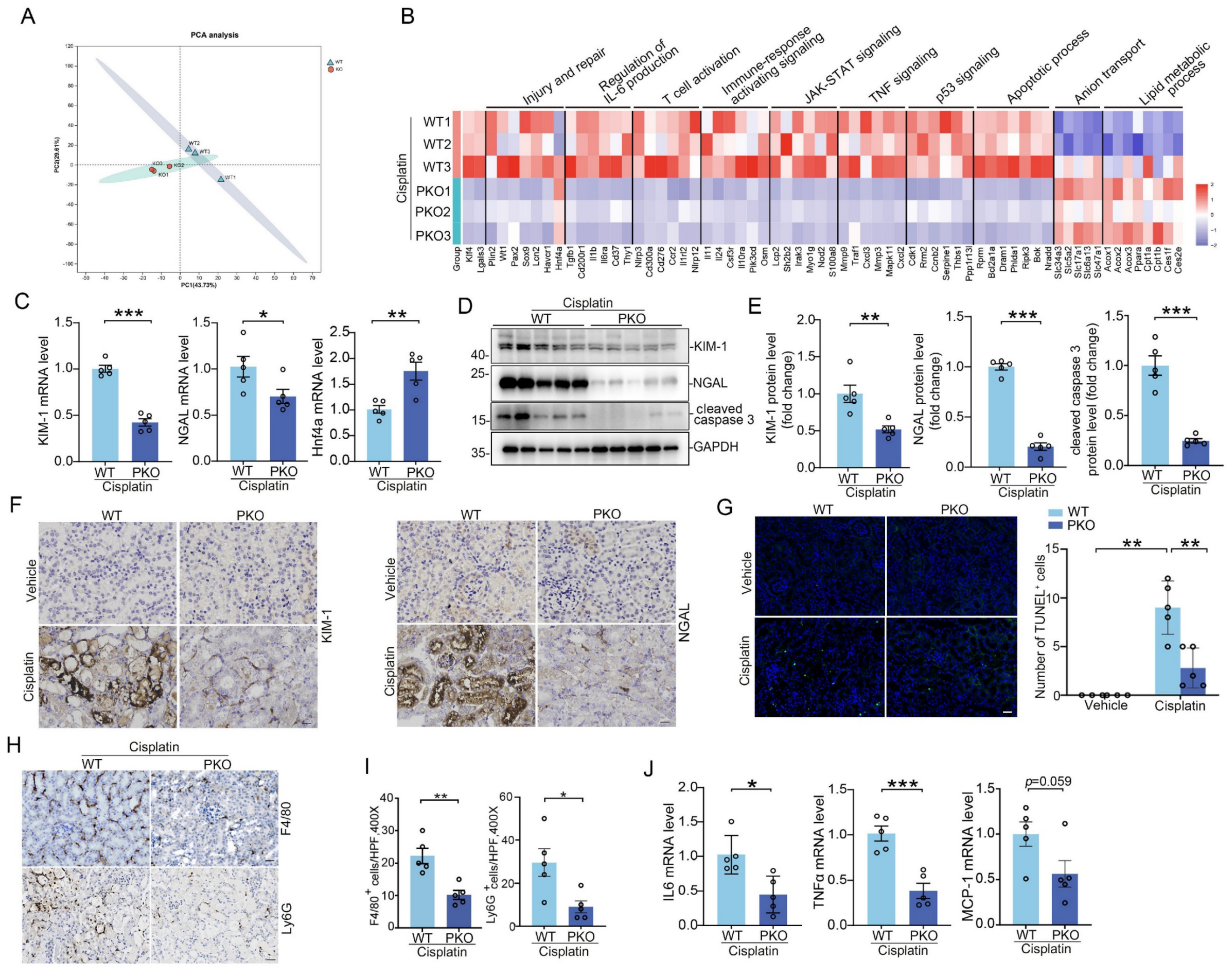
** Tubular KLF4 deficiency attenuates kidney injury, apoptosis and inflammatory response. (A)** Principal component analysis of global transcriptomics from WT and PKO kidneys following cisplatin challenge. **(B)** Heatmap of significant gene expression from WT and PKO kidneys with cisplatin exposure. **(C)** Renal mRNA expression levels of KIM-1, NGAL and Hnf4a in cisplatin-exposed kidneys from WT and PKO mice (n = 5). **(D)** Western blots for KIM-1, NGAL and cleaved caspase 3 in kidneys from WT and MKO mice after cisplatin injection at day 3. **(E)** Semiquantitative determination of protein abundance in (D) (n = 5). **(F)** Representative kidneys stained with KIM-1 and NGAL protein. Scale bar = 50 μm. **(G)** Representative images and quantification of TUNEL staining in kidney sections from WT and PKO mice with cisplatin nephropathy (n = 5). Scale bar = 50 μm. **(H)** Representative immunofluorescence staining for F4/80 and Ly6G in cisplatin-exposed kidneys from different groups as indicated. Scale bar = 50 μm. **(I)** Quantitative analysis for F4/80-positive macrophages and Ly6G-positive neutrophils in cisplatin-exposed kidneys among groups as indicated (n = 5). **(J)** The IL6, TNFa, and MCP-1 mRNA expression levels in WT and PKO kidneys following cisplatin treatment (n = 5). Data are presented as means ± SEM. *p˂0.05, **p˂0.01, or *** *p*˂0.001.

To confirm deletion of KLF4 in KLF4-PKO animals, immunofluorescence staining showing KLF4 was largely reduced in the kidney cortex from the knockouts following cisplatin injection (Figure 5K). Meanwhile, we isolated kidney cortex and measured protein and mRNA levels for KLF4 and Galectin-3 after cisplatin treatment. Compared to WT littermates, PKO mice exhibited reduced expression of KLF4 and Galectin-3 in the kidney cortical tissues (Figure 5L-M). Thus, KLF4 expression in PT cells accounts for at least half of all KLF4 expression in the cisplatin-induced injured kidney. Consistently, the protein levels of KLF4 and Galectin-3 were also significantly lower in PKO kidneys compared to WT kidneys in cisplatin-induced AKI model (Figure 5N-O). We then tested whether deleting KLF4 selectively within proximal tubular cells influences the development of cisplatin-induced kidney injury. At day 3 after cisplatin injection, all mice had marked renal tubular injury (Figure 5P). However, the PKO kidneys demonstrated a relatively preserved kidney structure, as confirmed by blinded injury scores (Figure 5P). KLF4 PKO mice had lower serum creatinine and BUN levels compared with WTs (Figure 5Q). To discern divergent gene expression programs of PKO versus WT kidneys, we performed transcriptomic analysis of kidney cortex following cisplatin at day 3. Principal components analysis showed a clear separation pattern between the WTs and PKOs (Figure 6A). Consistently, renal transcript levels for KLF4 and Lgals3 were significantly decreased in PKO groups compared to WT groups (Figure 6B). We then conducted GO enrichment analysis of DEGs and noted two recurring themes among the signaling cascades regulated by these DEGs: immune-response activating signaling and apoptotic process (Figure 6B). Levels of renal mRNA expression for genes encoding NGAL and KIM-1 were lower in PKOs compare to WTs. In contrast, the mRNA levels of proximal tubular cell preserved function marker HNF4a were increased in PKOs compared to WTs, which aligned with the results of the transcriptomic analysis (Figure 6C).

Furthermore, Western blot and semiquantitative analysis demonstrated that renal injury markers Kim-1, NGAL, as well as cell death marker cleaved-caspase 3, were reduced in kidney tissues (Figure 6D-F). Here again, TUNEL staining assays also confirmed the results of Western blot and RT-PCR analyses (Figure 6G). Galectin-3 is reported as an intrinsic activator driving inflammation. To investigate whether the deletion of KLF4 in proximal tubule could impact the inflammatory milieu of the kidney, we performed immunostaining on kidney tissues using antibody against F4/80 and Ly6G to specifically identify macrophages and neutrophils, respectively. Immunostaining revealed comparable baseline levels of F4/80^+^ macrophages and Ly6G^+^ neutrophils in WT and PKO mice (Supplementary Figure S9A-B). These cells were markedly elevated in cisplatin-injured kidneys of WT mice, whereas KLF4 deficiency significantly reduced their accumulation in cisplatin-treated PKO mice compared to WT controls (Figure 6H-I). In this regard, the mRNA levels of IL6, TNFα, and MCP-1 in the PKO kidney approximately 50% of WT values (Figure 6J).

**Figure 7:**
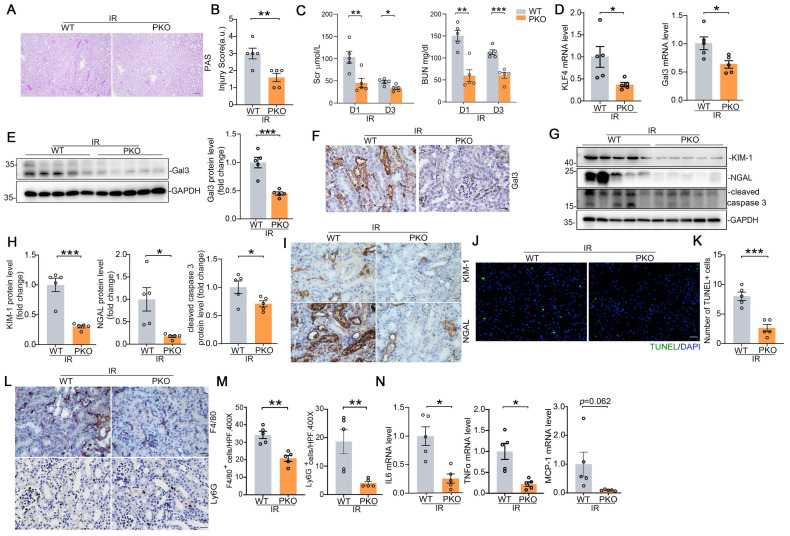
** KLF4 deletion in proximal tubular cells ameliorates IRI-induced kidney injury and inflammatory response. (A)** Representative images for PAS staining in kidneys among groups as indicated. Scale bar = 100 μm. **(B)** Kidney pathology scores in (A) (n = 5). **(C)** Serum creatinine and BUN levels in WT and PKO mice following IRI (n = 5). **(D)** Renal mRNA levels for KLF4 and Galectin-3 in IRI model (n = 5). **(E)** Western blot assay and semiquantitative analysis for Galectin-3 protein in IRI kidneys from different groups as indicated (n = 5). **(F)** Representative immunohistochemical staining for Galectin-3 protein in IRI kidneys among groups as indicated. Scale bar = 50 μm. **(G-H)** Western blot assay (G) and semiquantitative analysis (H) for KIM-1, NGAL and cleaved caspase 3 protein in IRI kidneys (n = 5). **(I)** Representative immunohistochemical staining for KIM-1 and NGAL protein in IRI kidneys among groups as indicated. Scale bar = 50 μm. **(J-K)** Representative images (J) and quantitative analysis (K) of TUNEL staining in the indicated groups (n = 5). Scale bar = 50 μm. **(L-M)** Representative immunochemical staining (L) and quantitative analysis of F4/80 and Ly6G (M) in IRI-induced kidneys from the indicated groups (n = 5). Scale bar = 50 μm. **(N)** Renal mRNA expression levels for IL6, TNFa and MCP-1 in IRI kidneys among groups as indicated (n = 5). Data are presented as means ± SEM. *p˂0.05, **p˂0.01, or *** *p*˂0.001.

To confirm these findings in a separate model of AKI, we employed IR-induced AKI model in which Galectin-3 was also largely localized in injured proximal tubule. Compared with WTs, PKO mice had significantly less tubular damage and lower BUNs and creatinines than WTs (Figure 7A-C). Renal mRNA expression for KLF4 and Galectin-3 were significantly lower in IRI-challenged PKO mice compared to WT controls (Figure 7D). Western blot and immunostaining analysis of Galectin-3 further confirmed qPCR results (Figure 7E-F). We then found PKO animals exhibited lower levels of injury markers KIM-1, NGAL, reduced protein levels of the apoptosis marker cleaved caspase 3, and fewer TUNEL positive cells compared to WT controls (Figure 7G-K). In addition, kidney inflammatory responses including inflammatory cell infiltration, as well as IL6, TNFα, and MCP-1 expression levels, were significantly reduced in the IR-injured kidneys of PKO mice compared to their WT control littermates (Figure 7L-N). In the IRI-induced kidney fibrotic model, PKO mice exhibited lower levels of fibrotic proteins compared to WT mice (Supplementary Figure S10A-D). Together, these data suggest that KLF4 in proximal tubular cells exacerbates cell death and inflammatory responses induced by cisplatin and IR potentially through the upregulation of Galectin-3.

### KLF4 inhibition with Kenpaullone attenuates cisplatin and IRI-induced acute kidney injury

**Figure 8:**
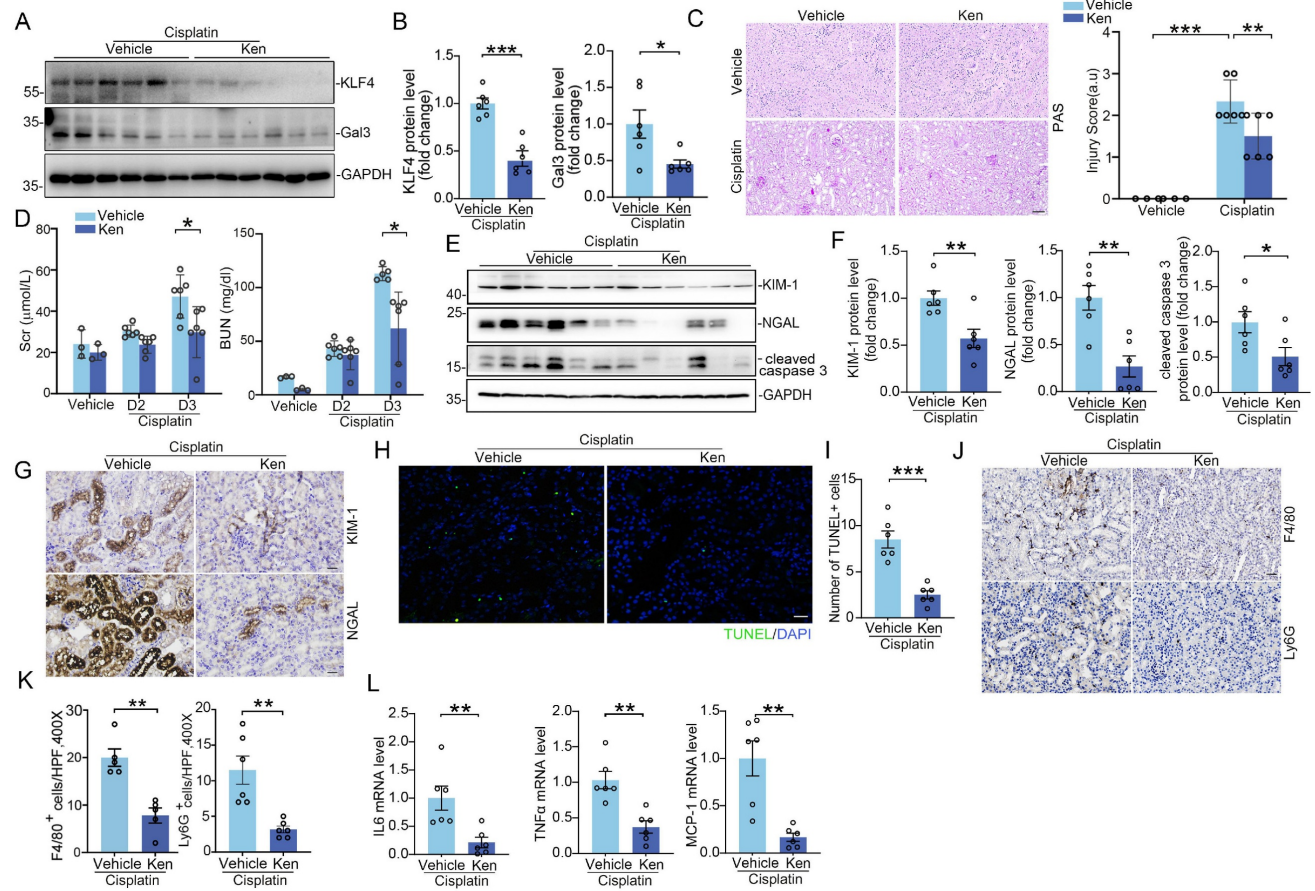
** Inhibition of KLF4 signaling with Kenpaullone attenuates cisplatin-induced acute kidney injury. (A-B)** Western blot assay **(A)** and semiquantitative analysis (B) for KLF4 and Galectin-3 protein in cisplatin-exposed kidneys among groups as indicated (n = 6). **(C)** Representative images for PAS staining in cisplatin-exposed kidneys and kidney pathology scores (n = 6). Scale bar = 100 μm. **(D)** Serum creatinine and BUN levels in groups as indicated (n = 6). **(E-F)** Western blot assay (E) and semiquantitative analysis (F) for KIM-1, NGAL and cleaved caspase 3 protein in cisplatin-exposed kidneys among groups as indicated (n = 6). **(G)** Representative images for KIM-1 and NGAL staining in cisplatin-exposed kidneys from vehicle and Kenpaullone-treated mice. Scale bar = 50 μm. **(H-I)** TUNEL staining (H) and quantification analysis (I) of kidney sections from vehicle and Kenpaullone-treated mice following cisplatin exposure (n = 6). Scale bar = 50 μm. **(J-K)** Representative immunofluorescence staining (J) and quantitative analysis (K) of F4/80 and Ly6G in cisplatin-treated kidneys from the indicated groups (n = 5). Scale bar = 50 μm. **(L)** IL-6, TNFa, and MCP-1 mRNA abundance in vehicle and Ken-treated kidneys following cisplatin exposure (n = 6). Data are presented as means ± SEM. *p˂0.05, **p˂0.01, or *** *p*˂0.001.

Based on these results above, we posited that blocking KLF4 signaling in renal tubular cells attenuates AKI through suppressing its downstream proteins Galectin-3 expression. To confirm whether pharmacologically blocking KLF4/Galectin-3 signaling could attenuate AKI, mice subjected to cisplatin-induced AKI models were treated with KLF4 inhibitor, Kenpaullone. After treatment with Kenpaullone, the protein levels for KLF4 and Galectin-3 were significantly reduced in the kidneys compared to those treated with vehicle (Figure 8A-B). By contrast, there was no difference in the protein expression of other KLF4 family members, including KLF5, KLF6, and KLF15 (Supplementary Figure S6C). In Kenpaullone-treated mice, severe pathological injuries in cisplatin-challenged kidneys, including the loss of brush border and tubular cell detachment, enlargement of tubular lumen and cast formation, were partially reversed (Figure 8C). Moreover, Kenpaullone administration could significantly improve severe kidney dysfunction and damage as evidenced by lower levels of BUN, creatinine, as well as renal proteins KIM-1, NGAL and cleaved-caspase 3 proteins compared to mice receiving vehicle injections (Figure 8D-G). Meanwhile, TUNEL staining assays showed a significant decrease in the number of positive tubular cells in the Kenpaullone-treated kidneys (Figure 8H-I). Moreover, inflammatory responses assessed by the quantification of F4/80^+^ macrophages and Ly6G^+^ neutrophils (Figure 8J-K and Supplementary Figure S11A-B), as well as mRNA levels for cytokines and chemokines, including IL6, TNFα, and MCP-1 (Figure 8L), revealed a significant reduction in inflammation in the Kenpaullone-treated kidneys compared to vehicle controls following by cisplatin exposure. Consistent with the effects of Kenpaullone in the cisplatin model, a comparable attenuation of kidney injury was observed in mice experiencing IR-induced AKI (Supplementary Figure S12A-E). Kenpaullone treatment did not induce hepatotoxicity in the mice (Supplementary Figure S11C). Therefore, these in vivo results demonstrate that inhibiting KLF4 signaling with Kenpaullone improves cell injury and death, and reduces inflammatory response, potentially through suppressing Galectin-3 transcription.

## Discussion

In this study, Galectin-3 was identified as a hub gene involved in the development and progression of various forms of AKI within failed repair or injured proximal tubules, as revealed by high-throughput single-cell transcriptomic analysis. By integrating an open-access database of transcription factor targets with genetic manipulation and pharmacological interventions in both *in vivo* and *in vitro* studies, we demonstrate that the transcription factor KLF4 positively regulates Galectin-3 transcription, promoting tubular cell death, kidney injury, and inflammatory responses in the context of AKI. Galectin-3, a detectable extracellular protein in biopsies and urine, may serve as a potential biomarker for patients undergoing AKI. This study offers novel mechanistic insights into the KLF4/Galectin-3 signaling as a shared pathogenic cascade in tubular cell injury and AKI. Targeting this pathway in tubular cells may hold promise as a potential therapeutic strategy for AKI.

Galectin-3 is a member of the galectin family, that can bind to b-galactoside sugars by either N-linked or O-linked glycosylation through their carbohydrate recognition domain 25, 26. While primarily located in the cytoplasm, Galectin-3 can also translocate to the nucleus and be secreted onto the cell surface and into biological fluids, including serum and urine, under disease conditions 27. Clinical research has found serum Galectin-3 levels potentially serve as a prognostic indicator of AKI and predicted mortality in sepsis 28. Among post-cardiac surgery patients, median serum and urine Galectin-3 levels on intensive care unit (ICU) admission were higher in AKI patients 29. Galectin-3 at the time of ICU discharge is associated with severity of AKI. Interestingly, preoperative serum Galectin-3 can also predict AKI after cardiac surgery, and may be used to augment risk information for patients at the highest risk of developing AKI and AKI severity after cardiac surgery 30. In this context, the present study overcomes the limitation of Galectin-3 being used for solely cardiac surgery- or sepsis-related AKI, thereby enhancing its clinical utility. It establishes a theoretical foundation and provides clinical validation for the use of Galectin-3 as a non-invasive 'damage' biomarker for kidney tubular injury across various types of AKI, in contrast with Scr, which primarily serves as a biomarker for glomerular filtration. Furthermore, previous studies have shown that higher serum and urinary Galectin-3 concentrations are with more severe kidney interstitial fibrosis and the progression of chronic kidney disease 31. Nevertheless, in this current study, the lack of these patient outcome data limits to assess the impact of elevated Galectin-3 on subsequent kidney function, chronic kidney disease or fibrosis.

The expression and role of Galectin-3 has been most frequently reported in immune cells 32. It can be expressed and secreted by monocytes, dendritic cells, and other immune cells, and it modulates immune and inflammatory responses, including the regulation of Th1 and Th2 responses 33-36. Extracellular Galectin-3 binds to cell-surface receptors such as TLRs and modulate cell adhesion, cell activation, and cell migration 37, 38. One of the fundamental roles for Galectin-3 is the recruitment of leukocytes during the inflammatory process 39. Galectin-3 is reported to regulate the secretion of cytokines and chemokines, promote polarization of macrophages toward inflammatory phenotype, and thereby modulate organ-specific and systemic inflammation 40, 41. Inhibition of Galectin-3 significantly reduced M1-type macrophage polarization while markedly increased M2-type in lung ischemia-reperfusion injury 42. Myeloid deletion of Galectin-3 reduced neutrophil recruitment, lung inflammation, and reduced lung fibrosis induced by chronic bleomycin 43. In contrast to previous reports suggesting that Galectin-3 primarily originates from immune cells, our results indicated that it was mainly located in kidney tubular cells, which possibly serve as the major source of Galectin-3. Although distal tubules express lower levels of Galectin-3 compared to PTs, we acknowledge that Galectin-3 in distal tubules may also contribute to AKI. We posited that Galectin-3 expressed and secreted by tubular cells attracted and activated immune cells, such as macrophages and neutrophils to provoke inflammatory responses during acute kidney injury. Moreover, in our previous reports and others, endothelial cells play a pivotal role in the inflammation of AKI through mechanisms such as upregulating adhesion molecule (ICAM-1, VCAM-1, P-selectin and E-selectin) 44, 45 and the IL-1 signaling pathway 7. The role of tubular cell-derived Galectin-3 in modulating endothelial cell behaviors warrants further research to better understand the progression of AKI. Nevertheless, a few studies reported anti-inflammatory role of Galectin-3 in the development and progression of organ specific inflammatory diseases 46, 47. For example, DC-derived Galectin-3 suppresses Th17 response *in vivo* by regulating IL-23 cytokine production 48. Galectin-3 stimulates activation of PI3K/Akt signaling pathway in macrophages and promotes their conversion in M2 immunosuppressive phenotype 49, 50. Endogenous Galectin-3 negatively regulates the production of IL-17 axis cytokines in dendritic cells stimulated with dectin-1 and TLR4 agonists, thereby inhibiting Th17 cell differentiation and development 48. These discrepancies may arise from differences in cell lineages or the experimental disease models used.

Although numerous studies have investigated the role and function of Galectin-3 in kidney diseases, the mechanisms regulating its expression, particularly in tubular cells during acute kidney injury remain unknown. To our knowledge, this is the first report detailing the effects of the pluripotent reprogramming factor KLF4-regulated galcetin-3 on tubular injury and inflammatory response in the kidney diseases. In this study, Galectin-3 promotes cell death not only through direct effects on tubular cells but also by provoking an influx of myeloid cells and stimulating the generation of cytokines with consequent acute kidney injury. Galectin-3 was reported to directly interact with anti-apoptotic protein Bcl-2, instead of bax, and promote cell apoptosis 51. It may also enhance pro-apoptotic signaling through JNK and ERK signaling pathways, as well as mitochondrial-dependent mechanisms 52, 53. Nevertheless, AKI engages multiple distinct regulated cell death pathways, including apoptosis, ferroptosis, necroptosis, pyroptosis, and cuproptosis. While this study specifically investigates tubular apoptosis, other forms of cell death remain under explored in the context of AKI. Further research is warranted to elucidate the regulatory role of KLF4/Galectin-3 signaling in modulating alternative tubular cell death programs, repair mechanisms, and proliferative responses following AKI.

KLF4 known as a Yamanaka factor, is responsible for cell reprogramming and pluripotency maintenance 54. It is highly expressed in various cell lineages and regulates a wide range of biological processes, including cellular proliferation, differentiation and self-renewal 55. The role and function of KLF4 have been studied in depth in kidney diseases. For example, KLF4 has been studied primarily in podocytes, where its expression is decreased in both animal models and humans exhibiting a proteinuria. The podocyte-specific loss of KLF4 leads to increased susceptibility to glomerulopathies 56. Interestingly, kidney biopsies with severe kidney dysfunction have lower glomerular KLF4 expression compared with those with more preserved kidney function. The reduced expression of KLF4 aggravates podocyte dysfunction in adriamycin nephropathy by increasing methylation at the nephrin promoter and decreasing methylation at the promoters of other epithelial markers. KLF4 reactivation could induce epigenetic changes sufficient to cause reversal of podocyte injury and long-lasting reduction of proteinuria 57. Besides its beneficial effects in podocytes, endothelial KLF4 also improves renal function and enhances the benefits of statins in ischemic acute kidney injury by decreasing inflammatory response 13. Meanwhile, endothelial KLF4 is necessary for maintenance of a quiescent glomerular endothelial phenotype and its loss increases susceptibility to complement activation and induction of prothrombotic and pro-inflammatory pathways 58. KLF4 also serves as a critical regulator of macrophage polarization, playing a key role in the attenuation of kidney injury 14, 59. Collectively, KLF4 seems appear to play a protective role in these cells during the development of kidney injury. Rather, the expression pattern of KLF4 in tubular cells is quite different; we and other research groups find that it is upregulated in injured tubular cells in both *in vitro* and *in vivo* studies 60, 61, contrasting with the decreased expression observed in endothelial cells and podocytes during disease conditions. Hence, the effects of KLF4 activation in tubular cells during AKI warrant scrutiny. Given that *in vitro* studies demonstrated cisplatin and H_2_O_2_ treatment induced KLF4 expression in a time-dependent manner, we posited oxidative stress might enhance KLF4 activation during AKI. Furthermore, we uncover that KLF4 is a potential key transcription factor that drives the development of kidney injury and the inflammatory response by binding to the Galectin-3 promoter and promoting its transcription, as demonstrated by a combination of bioinformatic analysis, ChIP, and luciferase assays. In addition to Galectin-3-mediated cell injury and apoptosis, KLF4 may contribute to AKI through regulation of other cellular pathways identified by the bulk RNA-seq analysis, including cell cycle, TGF-β, NF-κB, Notch and p53 signalings. Although research on the effects of KLF4 on kidney injury and the inflammatory response is limited, Fujiu et al revealed that collecting duct expression of KLF5 is essential for renal epithelial cell apoptosis, injury and expression of the chemotactic proteins S100A8 and S100A9, which recruited inflammatory monocytes to the kidneys and promoted their activation into M1-type macrophage 62. Contrary to its positive role in embryonic stem cells and induced pluripotent stem cells, increased expression of KLF4 suppresses axion regeneration of adult retinal ganglion cells, and reduces self-renewal of neural stem cells and promotes their differentiation into glia-like cells 63-65. The mechanisms by which KLF4 exerts cell lineage-specific discrepant effects on the evolution of kidney disease remain unclear. The actions of KLF4 on the pathogenesis of kidney disease may be context- and cell-dependent.

In this study, our results suggest that tubular Galectin-3 is a key pathogenic mediator that triggers cell apoptosis, injury and renal inflammation, which is transcriptionally regulated by KLF4. Blockade of the KLF4/Galectin-3 cascade by specific inhibitor protects against AKI in rodent model. Targeting this pathway may offer a novel therapeutic strategy for the clinical management of AKI.

## Supplementary Material

Supplementary tables and figures.

## Abbreviations

AKI: acute kidney injury
KLF4: Krüppel-like factor 4
Scr: serum creatinine
NGAL: neutrophil gelatinase-associated lipocalin
KIM-1: kidney injury molecule 1
PTs: proximal tubular cells
TNF-α: tumor necrosis factor alpha
NTS: nephrotoxic serum
AA: aristolochic acid
HK-2: Human renal proximal tubule cell line
FBS: fetal bovine serum
RIPA: radioimmunoprecipitation assay buffer
IHC: immunohistochemistry staining
ChIP: chromatin immunoprecipitation
FDR: false discovery rate
DEGs: differentially expressed genes
eGFR: estimated glomerular filtration rate
PI: propidium iodide
GO: gene ontology
HNF4a: Hepatocyte nuclear factor-4 alpha
RT-PCR: real-time polymerase chain reaction
MCP-1: monocyte chemoattractant protein-1
