# Supplementary material for: The KLF4/Galectin-3 cascade is a key determinant of tubular cell death and acute kidney injury
Source: Int J Biol Sci. 2025 Sep 20;21(13):5802–20. doi: 10.7150/ijbs.110790 (PMC12509911; doi:10.7150/ijbs.110790)
Supplement: Supplementary file 1 — Supplementary tables and figures. [file ijbsv21p5802s1.pdf]

Supplementary Materials

Supplementary Figure S1. ScRNA-sequencing analysis of kidney cell subpopulations in four acute kidney injury models.

(A) Integrated single-cell transcriptome maps of kidneys from acute kidney injury models induced by cisplatin, IR, AA, and NTS, respectively. Broken lines encircling proximal tubular cells (PTs). **(B)** Upset plots showing down-regulated DEGs between PT cell clusters from these four AKI models. The bottom-left barplot showing the total high-interacting genes for each model.

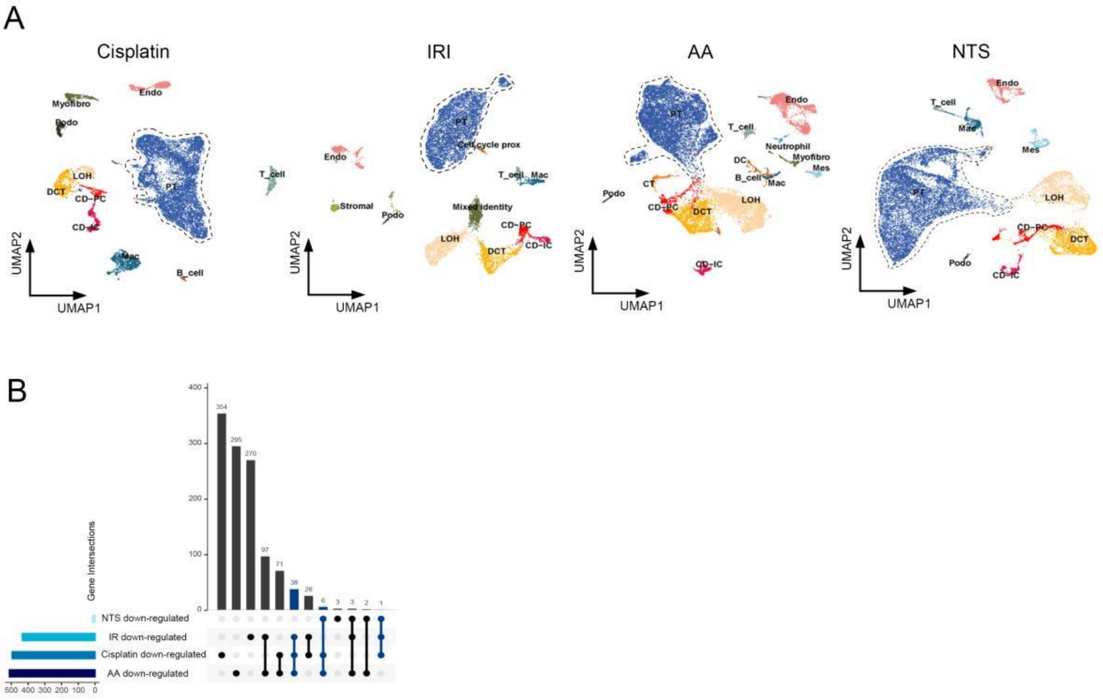

Supplementary Figure S2. *Lgals3* is predominant expressed in injured and/or failed repair PTs.

(A- D) UMAP plots of PT showing the expression of the indicated core genes, *Lgals3, C3, Anxa3, Cxcl1, Cxcl10, Cxcl2* and *Vcam1* identified through the protein-protein interaction (PPI) in the AKI models described above.

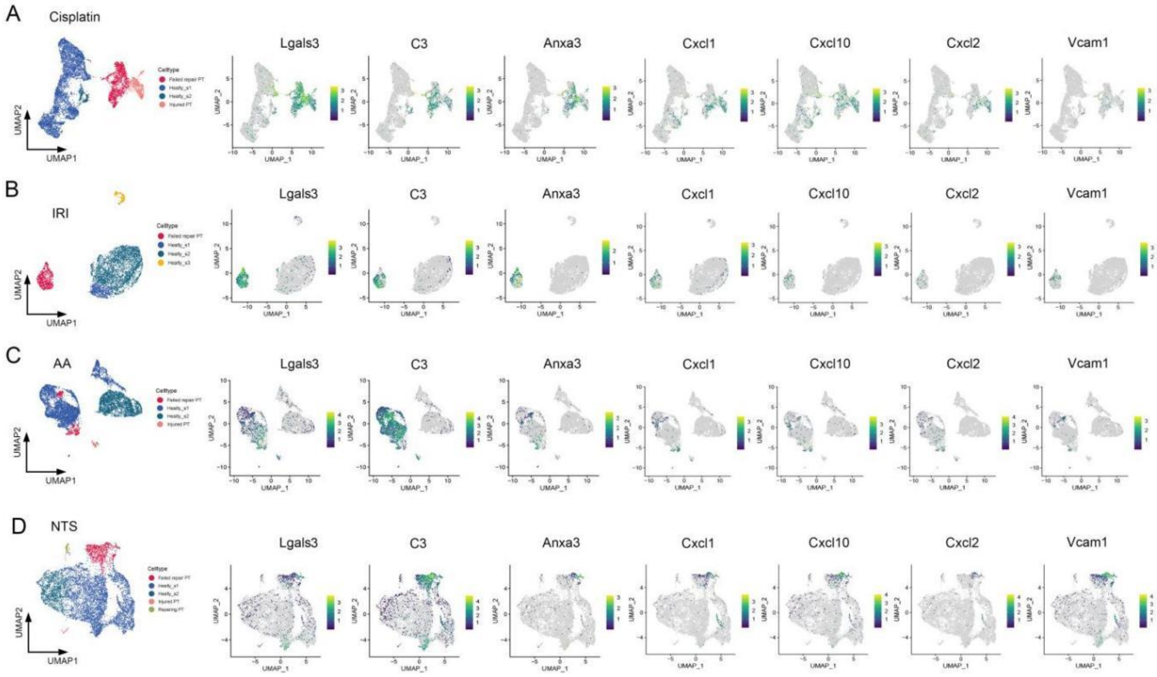

Supplementary Figure S3. (A) The expression of Lgals3 mRNA in different cell lineages across various AKI models. PT, Proximal Tubular cell; Podo, Podocyte; Endo, Endothelium; Mac, Macrophage; LOH, Loop of Henle; DCT, Distal Convoluted Tubule; CD-IC, Collecting Duct Intercalated Cell; CD-PC, Collecting Duct Principal Cell.

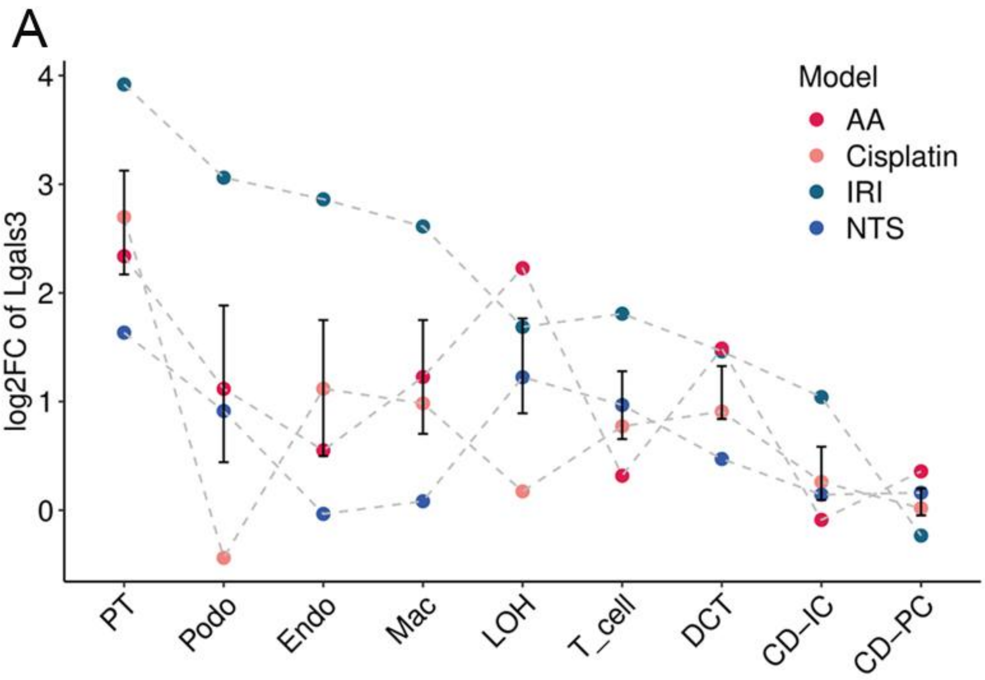

Supplementary Figure S4. (A) Representative immunohistochemical staining for F4/80 and Ly6G in sham mice treated with vehicle or GB1107. Scale bar = 50 μm. **(B)** Quantitative analysis for F4/80$^+$ macrophages and Ly6G$^+$ neutrophils between groups as indicated (n=3). **(C)** The plasma levels of AST and ALT in mice treated with either vehicle or GB1107 (n=3). AST, Aspartate Aminotransferase; ALT, Alanine Aminotransferase. Data are presented as means ± SEM.

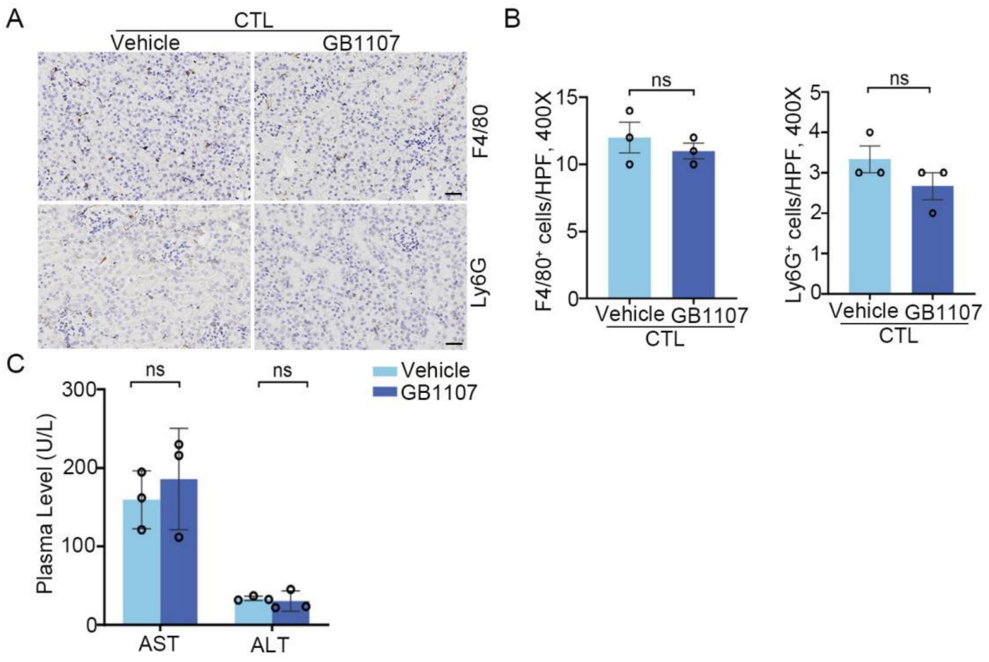

Supplementary Figure S5. Inhibition of Galectin-3 with GB1107 attenuates IR-induced acute kidney injury. (A) Representative images of kidney sections from vehicle- and GB1107- treated mice following IR-induced AKI. Scale bar = 50 μm. **(B)** Kidney pathology scores (n=4). **(C)** Blood urea nitrogen and serum creatinine. **(D)** Western blot for KIM-1, NGAL and Cleaved caspase3 between groups as indicated. **(E)** Semiquantification of KIM-1, NGAL and Cleaved caspase3 from (D) (n=4). Data are presented as means ± SEM. **p*<0.05, ***p*<0.01 or *** *p*<0.001.

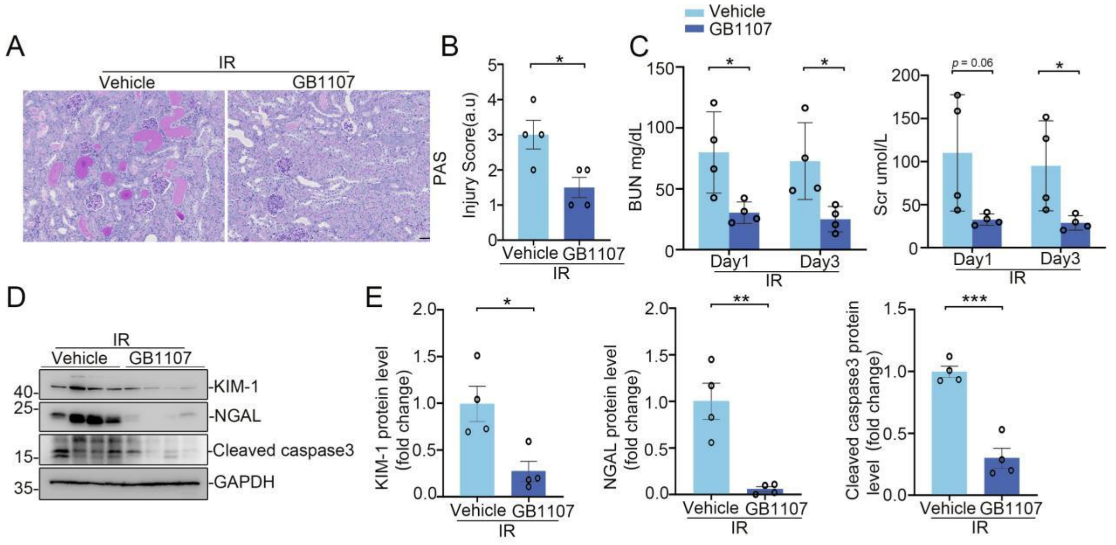

Supplementary Figure S6. (A-B) Western blot analysis of KLF5, KLF6, and KLF15 in HK2 cells treated with either vehicle/Kenpaullone (A) or vehicle/APTO-253 (B), followed by cisplatin exposure. **(C)** Western blot assay and semiquantitative analysis showing the abundance of KLF5, KLF6 and KLF15 protein in the mouse kidneys after cisplatin exposure (n=6).

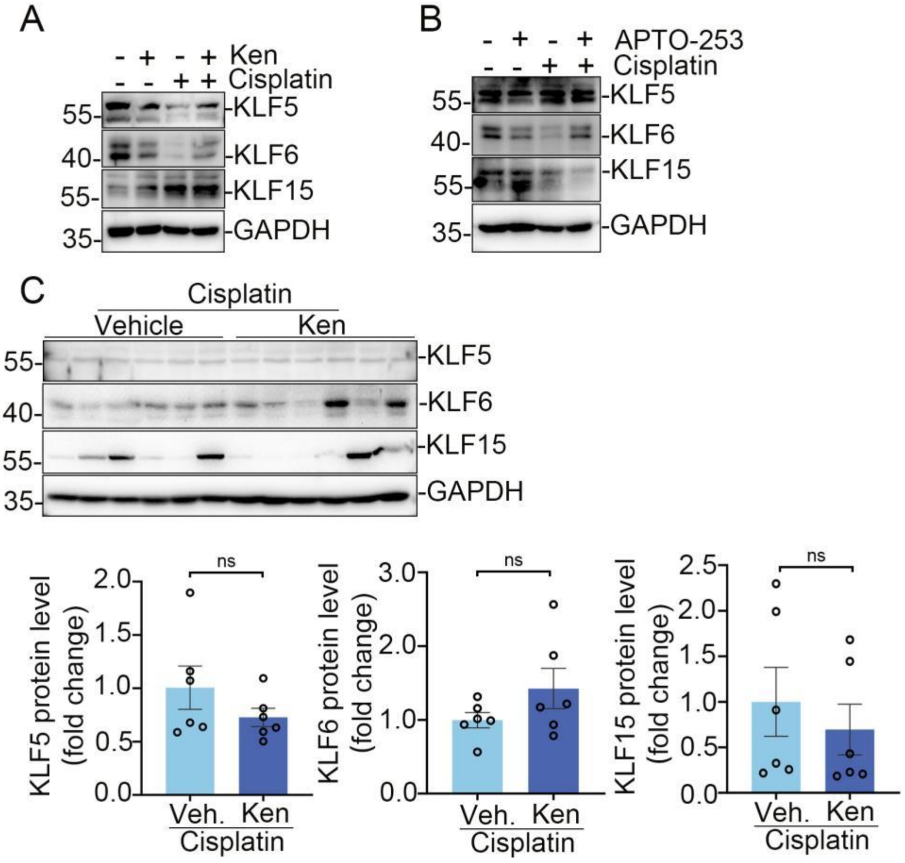

Supplementary Figure S7. (A-B) Linear regression of KLF4 (A) or LGALS3 **(B)** mRNA expression levels and eGFR in patients from Nephroseq database.

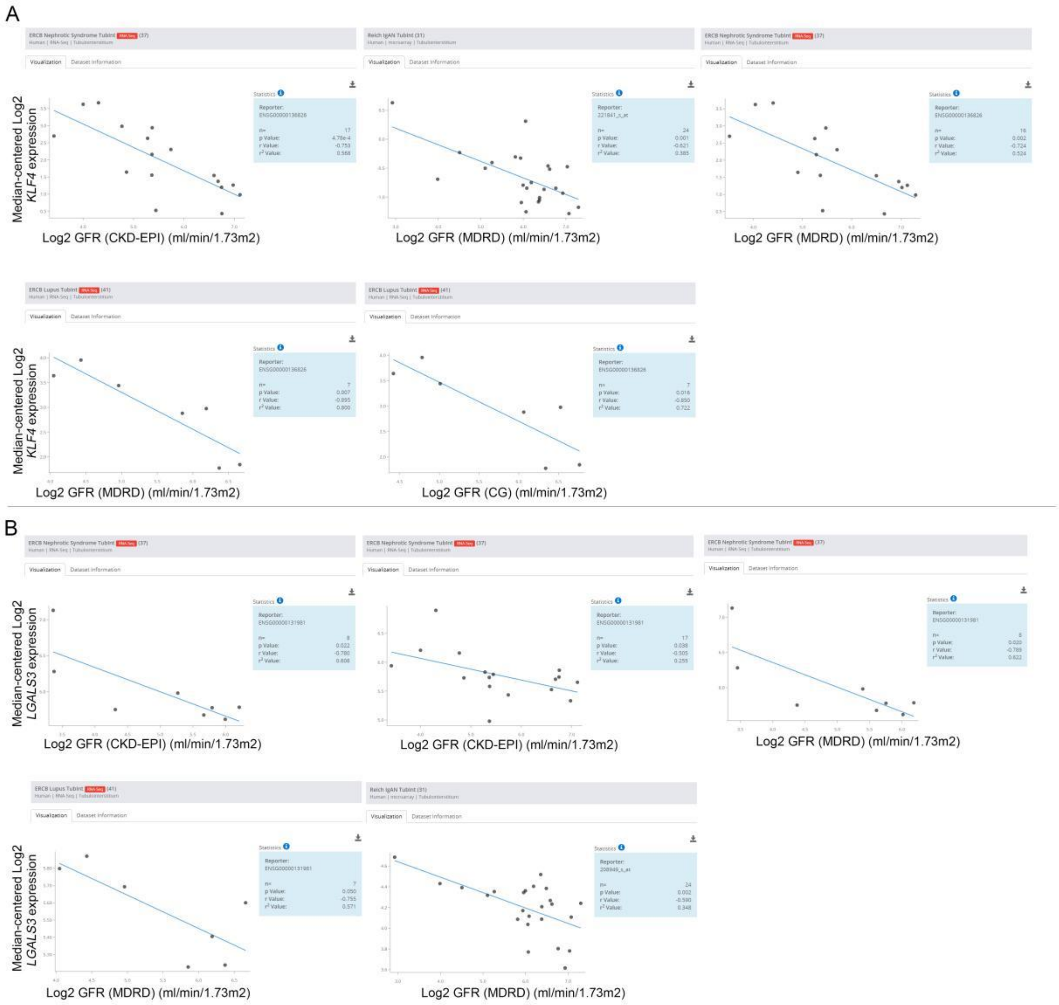

Supplementary Figure S8. Baseline parameters of WT and PKOs mice. **(A)** Body weight of WT and PKO mice at 3 and 6 months of age (n = 5). **(B)** Representative images for PAS staining in sham WT and PKO kidneys. Scale bar = 50 μm. **(C)** mRNA expression levels of AQP1, AQP2 and CDH16 in WT and PKO kidneys (n=4-5). mo, months; CDH16, cadherin 16; AQP, aquaporin. Data are presented as means ± SEM.

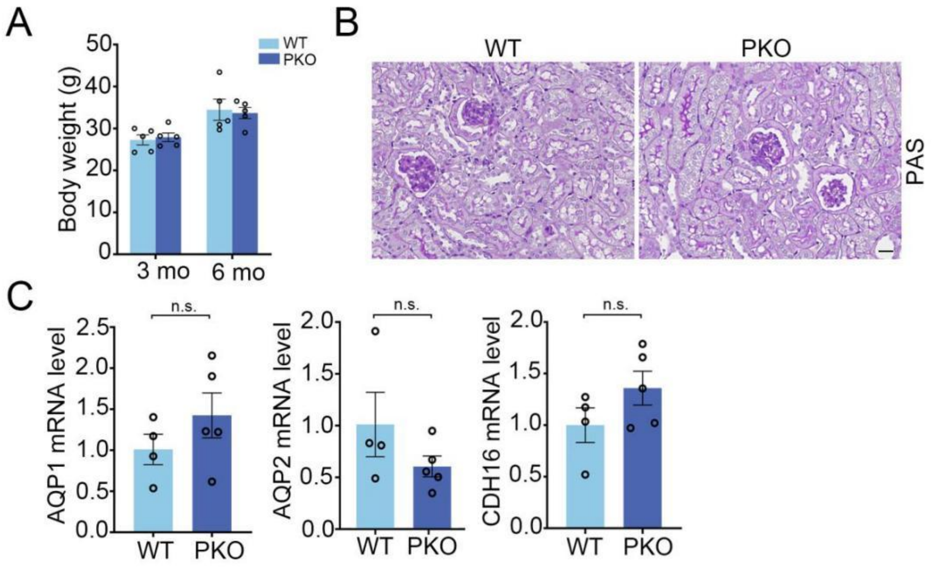

Supplementary Figure S9. (A-B) Representative immunohistochemical staining (A) for F4/80 and Ly6G, and quantitative analysis (B) of F4/80$^+$ macrophages and Ly6G$^+$ neutrophils in WT and PKO mice treated with vehicle (n=3). Scale bar = 50 μm.

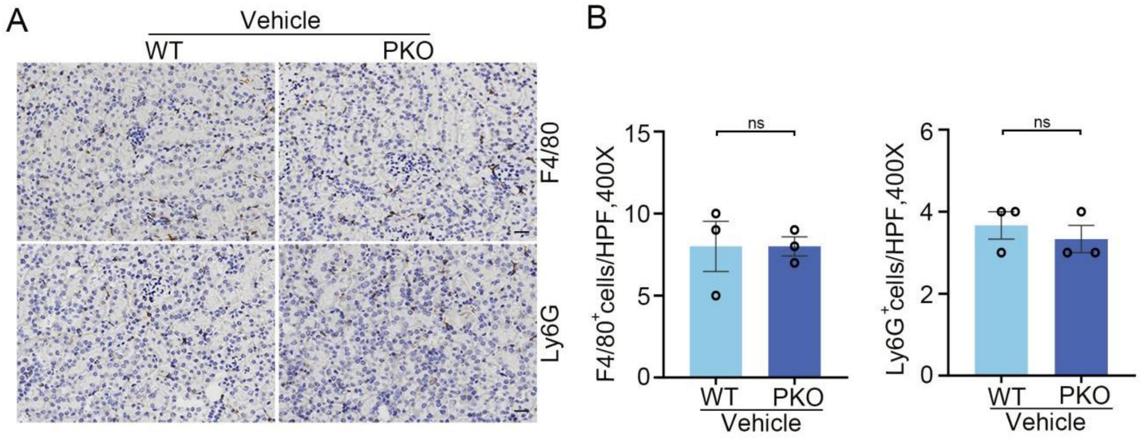

Supplementary Figure S10. KLF4 deletion in proximal tubular cells ameliorates IRI-induced kidney fibrosis. (A) Representative images for PAS and Masson staining in IRI kidneys among groups as indicated. Scale bar = 50 μm. **(B)** Graphic presentation showing the fibrotic area in IRI kidneys from the PKOs and control littermates (n=5). **(C-D)** Western blot assay (C) and semiquantitative analysis (D) for FN, Col-I and a-SMA protein in IRI kidneys from different groups as indicated (n=5). Data are presented as means ± SEM. **p*<0.05 or ***p*<0.01.

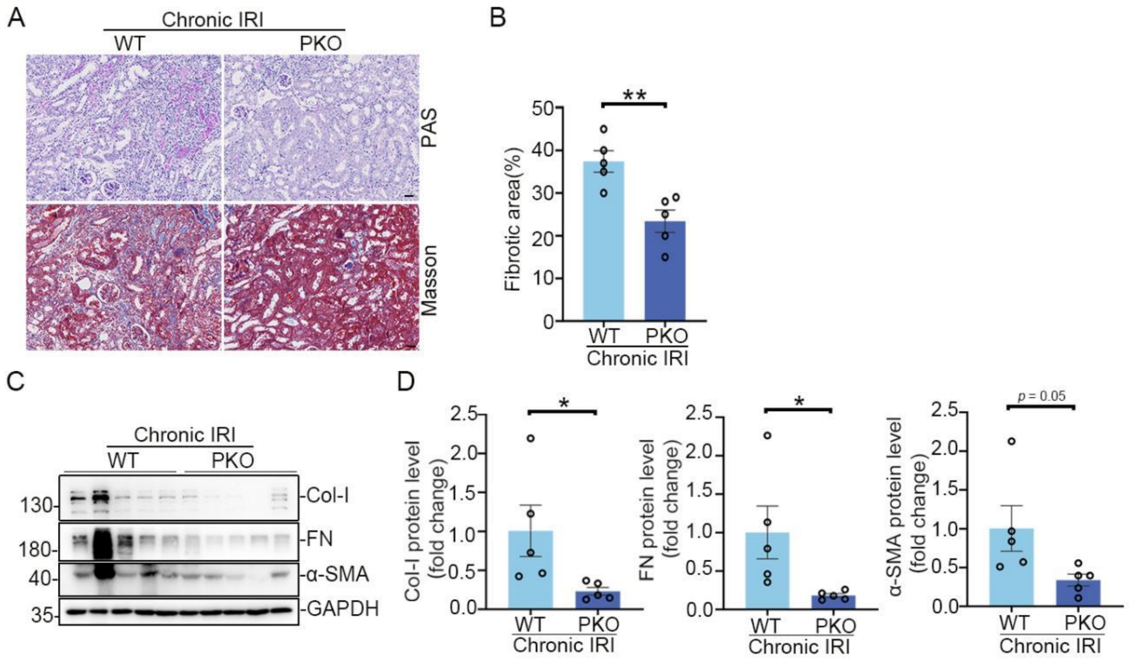

Supplementary Figure S11. (A-B) Representative immunohistochemical staining (A) for F4/80 and Ly6G, and quantitative analysis (B) of F4/80^{+} macrophages and Ly6G^{+} neutrophils in kidneys from mice treated with vehicle or Kenpaullone. Scale bar = 50 μm. **(C)** The plasma levels of AST and ALT in mice treated with either vehicle or Kenpaullone (n=3). AST, Aspartate Aminotransferase; ALT, Alanine Aminotransferase. Data are presented as means ± SEM.

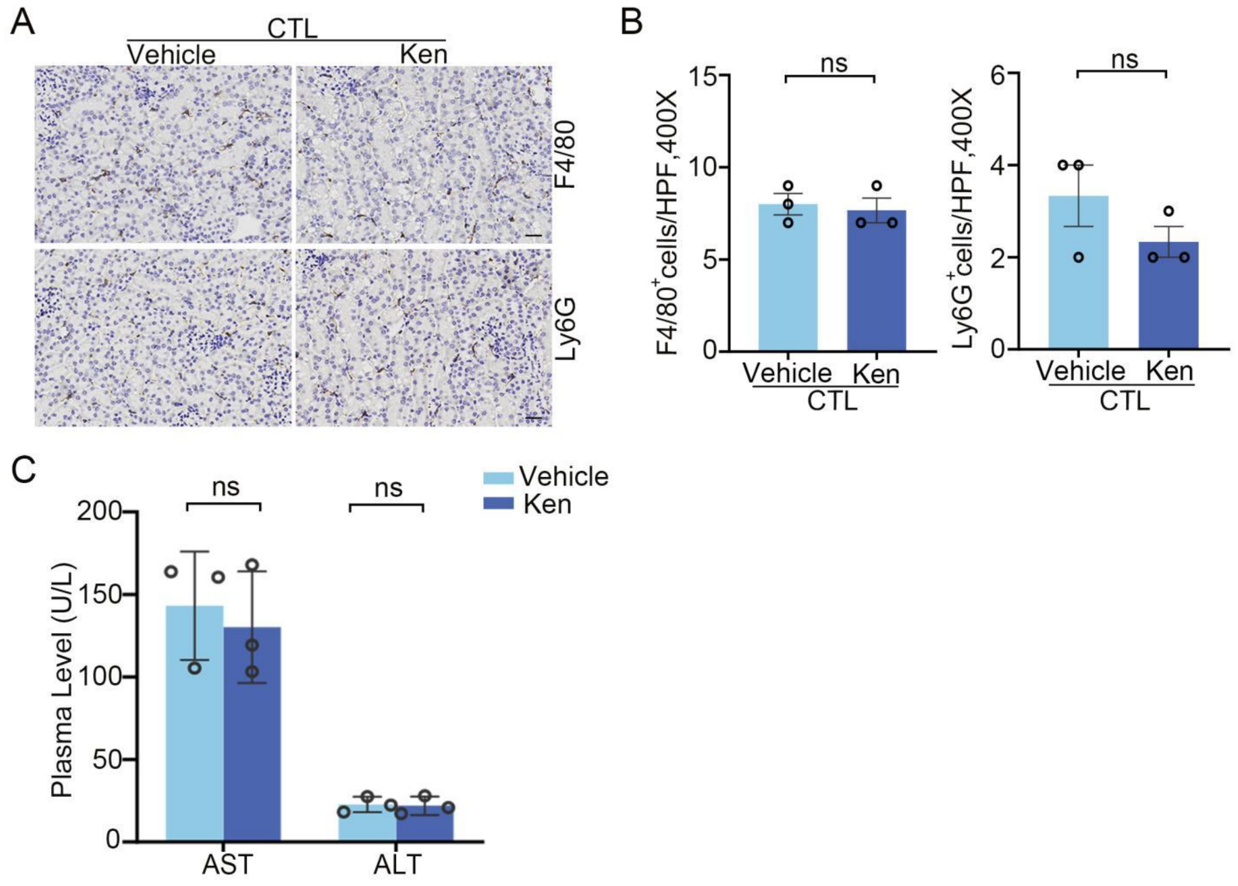

Supplementary Figure S12. Inhibition of KLF4 with Kenpaullone attenuates IR-induced acute kidney injury. (A) Representative images of kidney sections from vehicle- and Kenpaullone- treated mice following IR-induced AKI. **(B)** Kidney pathology scores (n=5). **(C)** Blood urea nitrogen and serum creatinine (n=5). **(D)** Western blot for KIM-1, NGAL and Cleaved caspase3 between groups as indicated. **(E)** Semiquantification of KIM-1, NGAL and Cleaved caspase3 from (D) (n=5). Data are presented as means ± SEM. **p*<0.05, ***p*<0.01, or *** *p*<0.001.

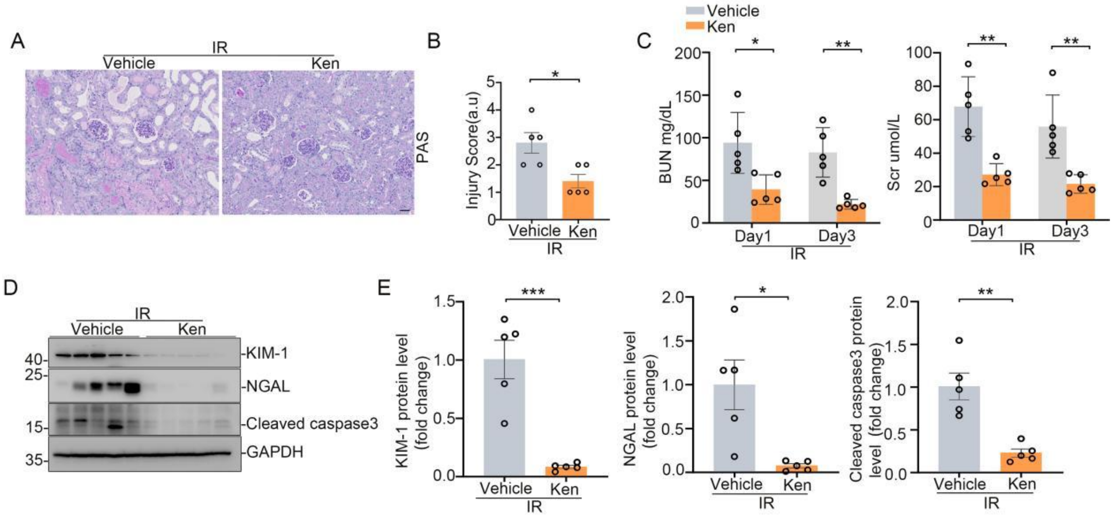

Supplementary Table S1. The primary antibodies were used in this study.

Primary antibody	Company	Cat.	Dilution rate
GAPDH	Proteintech	60004-1-Ig	1:10000
α-tubulin	Proteintech	11224-1-AP	1:10000
Galectin-3	Abcam	ab76245	1:10000
NGAL	Abcam	ab216462	1:1000
KIM-1	Abcam	ab78494	1:1000
cleaved caspase-3	Cell Signaling Technology	9664	1:1000
cleaved PARP	Cell Signaling Technology	5625	1:1000
KLF4	Santa Cruz Biotechnology	sc-393462	1:200

Supplementary Table S2. Clinical characteristics of healthy subjects and patients with AKI.

	**no AKI (n = 49)**	**AKI (n = 26)**
**Gender, n (%)**		
Female	16 (33)	12 (46)
Male	33 (67)	14 (54)
**Age (years)**		
Mean ± SEM	60.1 ± 2.1	65.3 ± 2.9
**BUN (mmol/L)**		
Mean ± SEM	5.1 ± 0.2	19.2 ± 1.4
**Scr (μmol/L)**		
Mean ± SEM	78.5 ± 2.3	227.7 ± 21.5
**eGFR (ml/min/1.73m^2)**		
Mean ± SEM	74.0 ± 2.8	23.4 ± 2.1
**Galetin-3 (ng/ml)**		
Mean ± SEM	197.7 ± 14.5	640.9 ± 114.9
**Etiology, n (%)**		
Kidney disease	/	6 (22)
Cardiac or vascular surgery	/	10 (39)
Septicopyemia	/	10 (39)
